# On the Ecological Significance of Phenotypic Heterogeneity in Microbial Populations Undergoing Starvation

**DOI:** 10.1128/spectrum.00450-21

**Published:** 2022-01-12

**Authors:** Monika Opalek, Bogna Smug, Michael Doebeli, Dominika Wloch-Salamon

**Affiliations:** a Jagiellonian University, Faculty of Biology, Institute of Environmental Sciences, Kraków, Poland; b Malopolska Centre of Biotechnology, Jagiellonian University, Kraków, Poland; c Department of Zoology, University of British Columbia, British Columbia, Vancouver, Canada; d Department of Mathematics, University of British Columbia, British Columbia, Vancouver, Canada; Broad Institute

**Keywords:** *S. cerevisiae*, bet hedging, evolutionary biology, mathematical modelling, quiescence, starvation

## Abstract

To persist in variable environments, populations of microorganisms have to survive periods of starvation and be able to restart cell division in nutrient-rich conditions. Typically, starvation signals initiate a transition to a quiescent state in a fraction of individual cells, while the rest of the cells remain nonquiescent. It is widely believed that, while quiescent (Q) cells help the population to survive long starvation, the nonquiescent (NQ) cells are a side effect of imperfect transition. We analyzed the regrowth of starved monocultures of Q and NQ cells compared to that of mixed, heterogeneous cultures from simple and complex starvation environments. Our experiments, as well as mathematical modeling, demonstrate that Q monocultures benefit from better survival during long starvation and from a shorter lag phase after resupply of rich medium. However, when the starvation period is very short, the NQ monocultures outperform Q and mixed cultures due to their short lag phase. In addition, only NQ monocultures benefit from complex starvation environments, where nutrient recycling is possible. Our study suggests that phenotypic heterogeneity in starved populations could be a form of bet hedging that is adaptive when environmental determinants, such as the length of the starvation period, the length of the regrowth phase, and the complexity of the starvation environment, vary over time.

**IMPORTANCE** Nongenetic cell heterogeneity is present in glucose-starved yeast populations in the form of quiescent (Q) and nonquiescent (NQ) phenotypes. There is evidence that Q cells help the population survive long starvation. However, the role of the NQ cell type is not known, and it has been speculated that the NQ phenotype is just a side effect of the imperfect transition to the Q phenotype. Here, we show that, in contrast, there are ecological scenarios in which NQ cells perform better than monocultures of Q cells or naturally occurring mixed populations containing both Q and NQ cells. NQ cells benefit when the starvation period is very short and environmental conditions allow nutrient recycling during starvation. Our experimental and mathematical modeling results suggest a novel hypothesis: the presence of both Q and NQ phenotypes within starved yeast populations may reflect a form of bet hedging where different phenotypes provide fitness advantages depending on the environmental conditions.

## INTRODUCTION

The survival of microbial populations depends on the individual cells’ ability to adjust their phenotype in response to challenging environmental conditions. Structured environments, ageing, and nutrient limitation have been identified as factors driving nongenetic heterogeneity, visible as multiple cellular phenotypes present in microbial populations (1–3). The switch to the quiescence phenotype in starved cells of the yeast Saccharomyces cerevisiae is a well-known phenomenon (4, 5): a fraction of the starved cells undergo specific molecular and cellular reprogramming and actively cease division when there is a lack of essential resources. As a consequence, in the stationary phase, genetically clonal, haploid yeast populations consist of a mixture of nonquiescent (NQ) and quiescent (Q) cells. In bacteria, growth advantage in stationary phase (GASP) phenotypes are widely studied, and they are well known to appear during long-term stationary phase (LTSP) (6, 7). Genetic changes in starved eukaryotes are much less studied. However, the evolution of GASP mutants has recently been reported within eukaryotic yeast after a 2-year long starvation (8). In particular, the evolution of GASP, transition to quiescence, and/or other spore-like cell types induced by starvation is of fundamental importance in medical microbial biology, since quiescence plays a crucial role not only in biofilm formation (9) but also in tumor formation (5).

The quiescent phenotype is complex, and its precise characterization is the subject of ongoing research (10–12). Q cells’ testing is also extremely challenging because, once they reenter the cell cycle, they are no longer in the quiescent state. And yet, studying quiescence in S. cerevisiae has the unique advantage of the possibility of obtaining the fractions of Q and NQ cells by centrifugation on a density gradient (13–16); Q cells are gathered in the denser, lower fraction, while the upper, less dense fraction predominantly consists of NQ cells. Quiescent yeast cells can be characterized by a thickened cell wall, dense vacuole, and an accumulation of storage materials like trehalose (5, 13, 17, 18). While starved, NQ cells are at various stages of the mitotic cell cycle and do not undergo the transition to Q cells. Moreover, NQ mother cells produce NQ daughters, while Q cells may transform to NQ cells at some unknown rate (19). As a consequence, NQ cells vary in internal organization and are more heterogeneous than Q cells. Differentiation into quiescence starts in growing yeast populations after the first signals of starvation, ca. 20 h after inoculation into a glucose-rich medium. For the common laboratory prototrophic S. cerevisiae strain S288C, the Q/NQ cell ratio is about 3:1 (13, 15, 20, 21).

While it is the whole population of Q and NQ cells that experience starvation, Q cells, with their adaptation to long-starvation survival, stress-tolerant viability, and higher recovery speed, are the ones responsible for population regrowth (13, 15, 22). Thus, evolved enrichment of Q cells in starved populations (up to 95%) results in a significant increase of the regrowth abilities after 22 days of starvation (20). Other research shows that after 4 weeks of starvation, Q cells exhibit 87% viability, while only 3% of NQ cells are still viable (counted as CFU) (13). Q cells also survive greater amounts of stress, such as temperature (13, 15) and toxins (including antifungals) (9). The regrowth abilities of Q and NQ cells were checked separately after culture fractionations; however, in most of the experimental setups, stress was applied to the unseparated stationary-phase population consisting of certain mixtures of Q and NQ cells (4, 14, 15, 23).

The Q/NQ population balance can be affected by many factors (22). It has been shown that the Q/NQ ratio can be modified by selection to some extent; however, both Q and NQ cells appear to always be present in stationary populations (14, 20, 24). This raises the important question of how the Q/NQ balance evolves in various ecological scenarios. Because of the clear advantage of Q cells in stress survival, the presence of NQ cells could be an inevitable by-product of cellular physiology, with no particular adaptive significance. For example, it has been hypothesized that replicative ageing is a factor determining the transition to quiescence: the presence of NQ cells in a starved population would simply reflect the inability of old cells to enter quiescence (23). However, recent research using more advanced laboratory techniques has questioned this interpretation (15). Alternatively, the presence of both Q and NQ phenotypes within a starved population may reflect some form of bet hedging, where different phenotypes provide fitness advantage depending on the environmental conditions (25–28).

**FIG 1:**
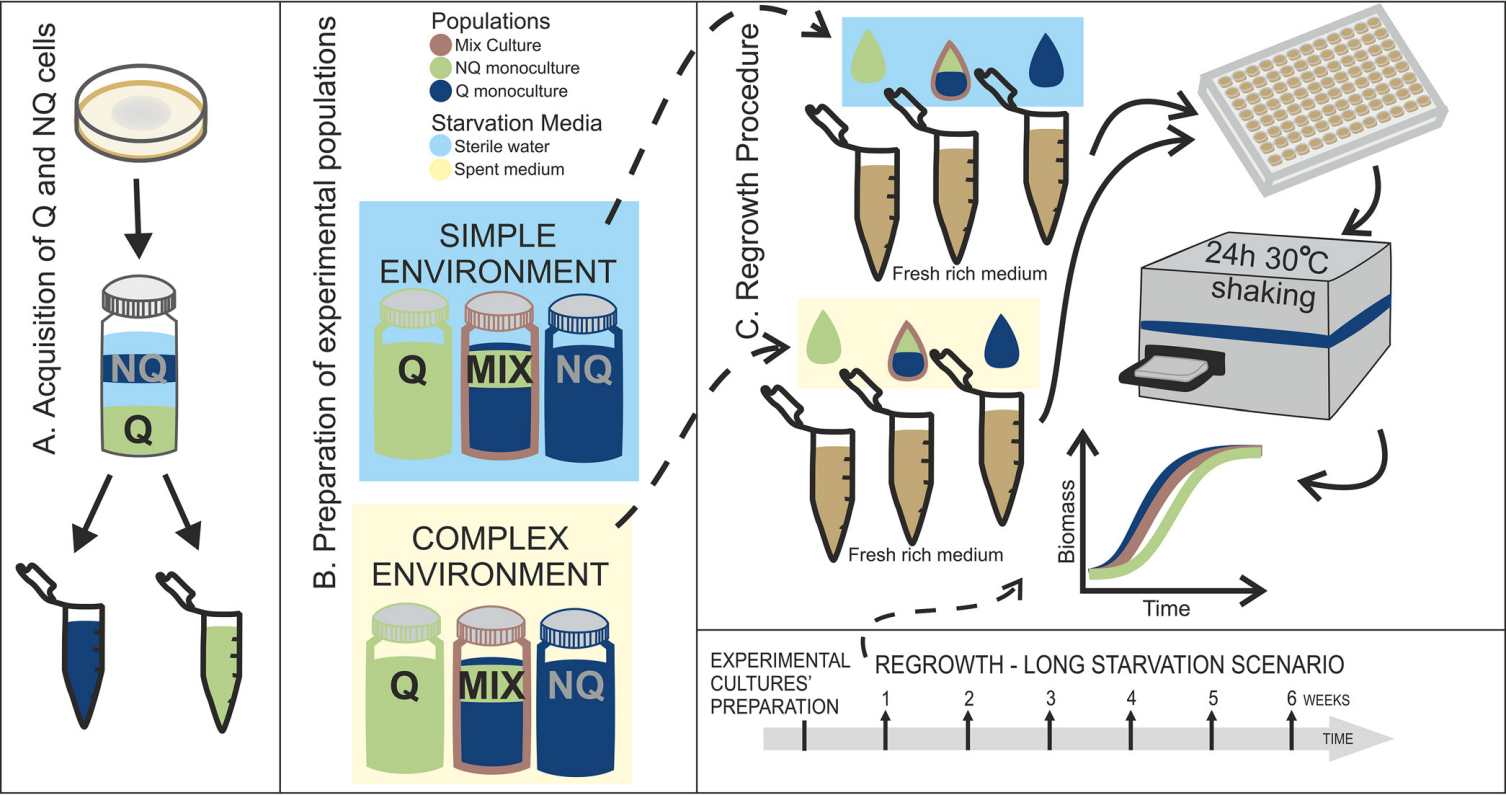
Schematic representation of experiments performed. (A) After inoculation, population was grown on an agar YPD plate for 4 days, after which Q (navy) and NQ (green) cells were separated by centrifugation on a density gradient. (B) Six types of experimental populations were prepared, and each type was prepared in five repetitions, giving 30 independent cultures altogether. First, all Q and NQ cells acquired by fractionation procedure were merged and diluted to equal ODs. Next, mixed cultures were prepared by mixing Q and NQ cells in 3:1 proportions. Then, cells were pelleted and resuspended in a starvation medium: sterile water for the simple environment (blue background) and spent medium for the complex environment (beige background). (C) To assess starvation survival, we took samples of experimental cultures weekly. Cells were pelleted and resuspended in fresh rich medium. Then, samples were loaded into 96-well plates and placed into the plate reader for regrowth. The samples were incubated for 24 h at 30°C with shaking, and OD measurements were taken every half hour. OD measurements were recalculated into biomass and relative biomass. Based on experimental data, the model parameters were fitted.

Here, we use population-level experiments to shed light on the adaptive significance of the Q/NQ cell ratio in yeast. We test whether populations composed entirely of Q cells (Q monoculture) have an ecological advantage over natural populations (mixed culture with Q and NQ cells in a 3:1 ratio), as well as over NQ monocultures. These mixed cultures imitate the naturally occurring Q/NQ cell ratio in starved laboratory strain S288C and are therefore taken as a reference point. We monitor the starvation survival of experimental cultures weekly via regrowth experiments (Fig. 1; see Materials and Methods for details). We describe the populations’ growth curves after various starvation lengths—a long-starvation scenario lasting from 1 to 6 weeks and preceded by an additional environmental stress, i.e., freezing, and a short-starvation scenario lasting 4 days. We analyze the impact of the environment during long starvation, where cells are suspended either in sterile water (simple environment) or spent medium (complex environment). Finally, we develop a mathematical model to explain our experimental results and to predict ecological outcomes.

Our experiments show that Q monocultures regrow relatively better than mixed cultures if the starvation phase is long enough and that this advantage diminishes after a longer regrowth time. We use the mathematical model to assess possible reasons for this advantage. The model supports the notion that the ecological advantage of Q monocultures is due to the lower death rate of Q cells during starvation and to shorter lag times of Q cells after starvation periods longer than 1 week. We also demonstrate that, due to nutrient recycling, NQ monocultures do relatively better in complex than simple starvation environments. Finally, we hypothesize based on the model and confirm experimentally that NQ monocultures can regrow faster than Q monocultures when the starvation period is very short. We conclude that the presence of both quiescent and nonquiescent cells could be advantageous for population survival in fluctuating environments because Q cells survive long starvation better and NQ cells restart division faster if the starvation period is short. Moreover, when there is a possibility of nutrient recycling in complex starvation environments, NQ cells may suffer less from unfavorable environmental conditions than from simple environments. This supports the hypothesis that the existence of both cell types in natural populations could be a form of bet hedging rather than the effect of imperfect transition into quiescence.

## RESULTS

### Long starvation in the simple environment. (i) Experimental data show that the advantage of Q monocultures is dynamic.

**FIG 2:**
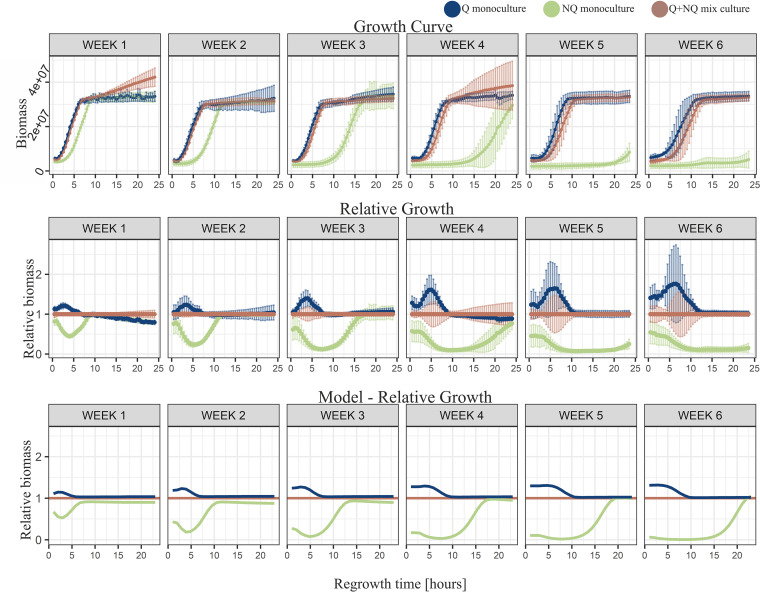
Simple environment results. Results from starvation in the simple environment. Sequential vertical graphs illustrate the results for regrowth after the indicated length of starvation in weeks (1 to 6). The regrowth time (in hours) illustrates time elapsed after placing samples from starved experimental cultures into fresh medium for regrowth. Points illustrate the averaged results from five repetitions (except for weeks 5 and 3 for NQ monoculture, where there were four repetitions each), and error bars show standard deviations. The Q (navy) and mixed (brown) cultures displayed similar growth curves until the 3rd week of starvation. When starved for 4 weeks or longer, the growth curve of the Q monoculture increased more sharply, reaching the flat shape of stationary phase earlier. The growth curve of the NQ monoculture (green) after the 1st week of starvation was the flattest, and it reached stationary phase later than both mixed and Q cultures.

A higher proportion of stress-resistant Q cells should provide better population survival during starvation, which can be measured as exponential biomass increase after the lag phase in a fresh growth medium. Accordingly, Q monocultures were expected to synchronously restart division soon after nutrient restoration and reach stationary-phase density before other cultures. Indeed, at the beginning of the regrowth procedure after the 1st week of starvation, Q monocultures had a biomass advantage over mixed cultures (after 2 h of regrowth, the average biomass of Q cultures was 8.22 × 10^6^ cells and the average biomass of mixed cultures was 6.85 × 10^6^ cells; *P* = 0.0005) (Fig. 2; Fig. S10 in the supplemental material). And yet, the advantage of Q monocultures was small, and during further weeks of experiments, it was not significant (for differences after 2 h of regrowth, significance was as follows: 2nd week, *P* = 0.33; 3rd week, *P* = 0.129; 4th week, *P* = 0.177; and 5th week, *P* = 0.404) except for the 6th week (*P* = 0.037) (Fig. S10).

### (ii) Experimental data show that NQ monocultures regrow more slowly.

**FIG 3:**
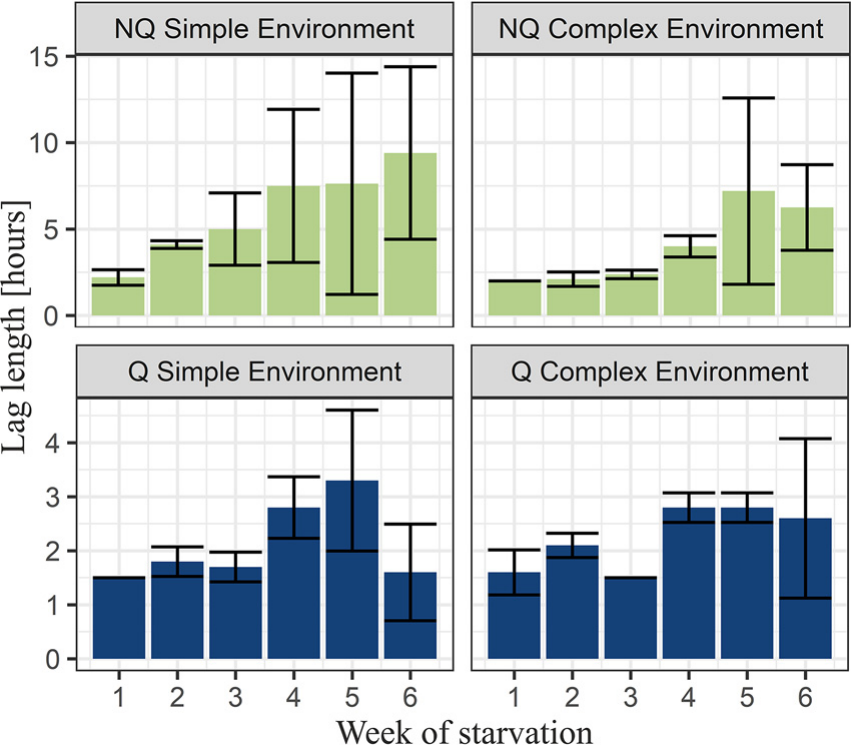
Average lag phase lengths of experimental monocultures during starvation. Bars represent standard deviations. An increased length of the lag phase was evident for NQ monoculture, and it was especially visible in cultures from the simple environment. Meanwhile, for Q monoculture, the lag phase length was almost constant throughout the whole starvation time.

The increase in biomass of NQ monocultures was slower than that of the other experimental populations (Fig. 2). The significant disadvantage was already visible after the 1st week of starvation, where initially NQ monocultures had lower biomass than the other cultures (for differences after 2 h of regrowth [average biomass of NQ cultures = 4.39 × 10^6^ cells], significance [*post hoc* Tukey test] was as follows: for NQ cultures compared to Q cultures [NQ-Q], *P* = 1.31 × 10^−8^, and for NQ-mixed, *P* = 1.74 × 10^−6^) (Fig. S10) and only reached the biomass of Q cultures and mixed cultures after 10 h of regrowth (Fig. 2, 10 h regrowth) (average biomass of Q cultures = 3.27 × 10^7^ cells, average biomass of mixed cultures = 3.32 × 10^7^ cells, and average biomass of NQ cultures = 3.29 × 10^7^ cells; *P* > 0.05 for both NQ-Q and NQ-mixed comparisons) (Fig. S10). Also, the lag phase of NQ monocultures was longer than those of the other experimental populations (Fig. 3; Table S1). The biomass differences between NQ and other cultures increased with starvation time. During further weeks of starvation, NQ monocultures needed more and more time to reach stationary-phase density, exceeding 24 h after the 4th week of starvation (Fig. 2).

We compared lag phase lengths for Q and NQ monocultures starved in simple environments. The length of the lag phase increased with starvation time (*P* = 3.58 × 10^−8^) (Fig. 3; Tables S1 and S2, Fig. S9). While at the beginning of the experiment, the lag phase was on average 1.5 h for Q and 2.2 h for NQ monocultures, the lag phase of NQ monocultures increased to an average of 9.4 h after the 6th week of starvation. The increase of the lag phase of Q monocultures was much lower than that of NQ monocultures (*P* = 0.0003) (Table S2), and its length was, on average, 1.6 h after 6 weeks of starvation.

### Analysis of model predictions.

In order to shed light on possible reasons for the advantage of Q monocultures over mixed cultures and on how this advantage depended on the length of starvation and regrowth time, we used a mathematical model that tracked the ratio of Q and NQ cells over time, as well as the concentrations of limiting nutrients (see the supplemental material for a detailed description of the model). The model reproduced the experimental results from the simple starvation environment: (i) the advantage of Q monocultures over other cultures increased with the starvation time, and (ii) Q cells’ advantage was noticeable at the beginning of regrowth, but with an increasing length of regrowth, this advantage decreased and finally disappeared (Fig. 2C).

The model suggests that two factors drive those results: the death rate during starvation and the lag length (if they were equal for Q and NQ cells, both cell types would follow the same starvation and regrowth dynamics). In particular, the dependence of the relative biomass of different culture types on the regrowth time resulted from the fact that Q cells had a shorter lag length than NQ cells (Fig. S6A). In contrast, the dependence of the relative biomass on the starvation time could result from either (i) a higher death rate for NQ cells than for Q cells during starvation (Fig. S6B) or (ii) lag lengths consistently growing with the starvation time (Fig. S6C).

Based on modeling, we also predicted that nutrient recycling is a potentially important factor influencing survival during starvation. The model suggested that NQ cells should be the ones that would benefit from a more complex starvation environment. Nutrient reusability helped NQ monocultures regardless of their lag time: the model yielded analogous results even if both Q and NQ cells had no lags during regrowth (Fig. S6D). This suggested that it was the nutrient recycling that drove the difference in NQ survival in the two environments.

### Long starvation in the complex environment: experimental data show that nutrient recycling is crucial for NQ cells’ survival.

**FIG 4:**
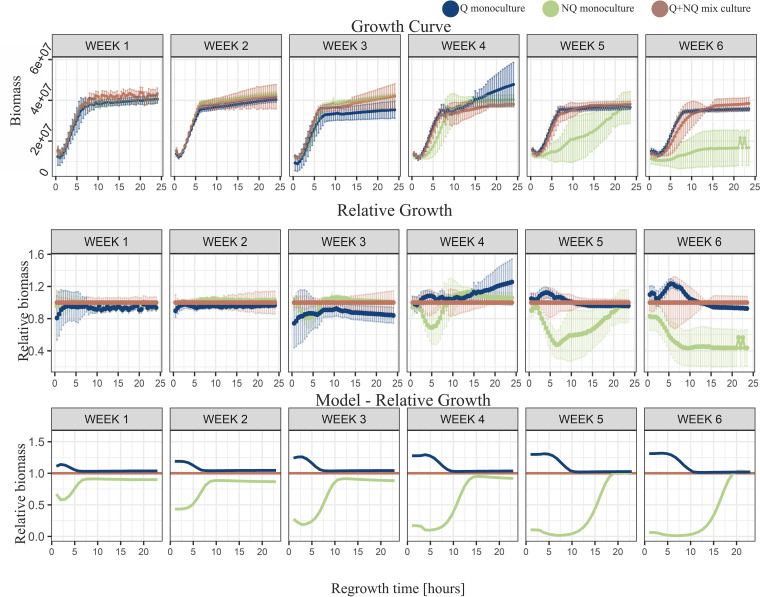
Complex environment results. Results from starvation in the complex environment. Sequential vertical graphs illustrate the results for regrowth after the indicated length of starvation in weeks (1 to 6). The regrowth time (in hours) illustrates time elapsed after placing samples from starved experimental cultures into fresh medium for regrowth. Points illustrate the averaged results from five repetitions (except for week 6 for NQ monoculture, where there were three repetitions), and error bars show standard deviations. The growth curves of all starved populations were similar until the 3rd week of starvation. When starved for 4 weeks or longer, the growth curves of NQ monocultures gradually flattened. Q and mixed cultures displayed similar shapes of growth curves throughout the whole starvation time.

To test the model predictions, we repeated our long-starvation experiment in the complex environment, where nutrient recycling was possible. The experimental results revealed that there was no significant difference between populations at the beginning of regrowth or up to the 5th week of starvation (after 2 h of regrowth, the *P* value was >0.05 for all NQ-Q, NQ-mixed, and Q-mixed comparisons) (Fig. S11). Up to the 2nd week of starvation, NQ monocultures’ regrowth was similar to Q and mixed cultures’ regrowth (Fig. 4), reaching the same biomass after sufficient regrowth time (for maximal density after ∼5 h of regrowth at the 1st week, the average biomass of Q cultures was 3.04 × 10^7^ cells, the average biomass of NQ cultures was 3.23 × 10^7^ cells, and the average biomass of mixed cultures was 3.21× 10^7^ cells; *P* > 0.05 for all NQ-Q, NQ-mixed, and Q-mixed comparisons). After further weeks of starvation, the differences in regrowth efficiency between NQ monocultures and mixed cultures on the one hand and Q monocultures and mixed cultures on the other hand gradually increased. After the 4th week of starvation, NQ monocultures reached stationary-phase density within 10 h of regrowth, and a week later, they needed almost 24 h to reach the same biomass (Fig. 4). In terms of length of regrowth, the same pattern could be observed as described previously—with increasing regrowth time, differences between populations progressively decreased and finally disappeared when regrowth time was long enough for all cultures to reach stationary-phase density (Fig. 4; Fig. S7 and S8).

**FIG 5:**
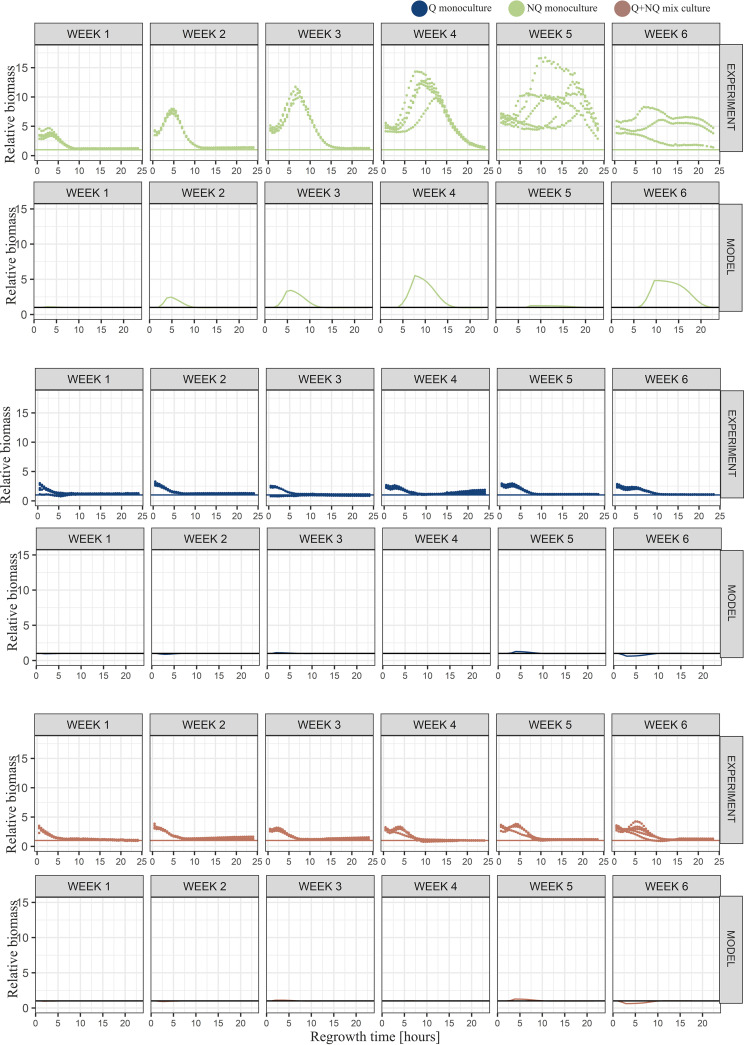
Complex versus simple environment. Starvation environments’ impacts on cultures’ starvation survival. Sequential vertical graphs illustrate the results for regrowth after the indicated length of starvation in weeks (1 to 6). Regrowth time (in hours) illustrates time elapsed after placing samples from starved experimental cultures into fresh medium for regrowth. Relative biomass was calculated as the ratio of biomass of a given cell type culture from the complex environment to the biomass of the same cell type culture from the simple environment (dots). The biomass of a culture in the simple environment equals 1 (solid line). NQ monocultures survived relatively better when starved in the complex than in the simple environment. Q and mixed cultures benefitted slightly at the beginning of regrowth (up to 10 h) when starved in the complex environment.

Direct comparison of the simple and complex starvation environments revealed that the biomass of NQ monocultures could even be 11 times higher (4th week of starvation, regrowth time from 8.5 to 11.5 h) when starved in the complex environment (Fig. 5). The model results followed the general pattern of the experiments (except for NQ monocultures in the 5th week, probably due to an unusually long experimental lag phase in the complex environment) (Table S1), although some quantitative differences might also have been caused by large variance in experimental data. The regrowth abilities of Q monocultures and mixed cultures were influenced by starvation medium to a lesser extent (Fig. 5).

### Short-starvation scenario: NQ monoculture has the biomass advantage in short starvation.

**FIG 6:**
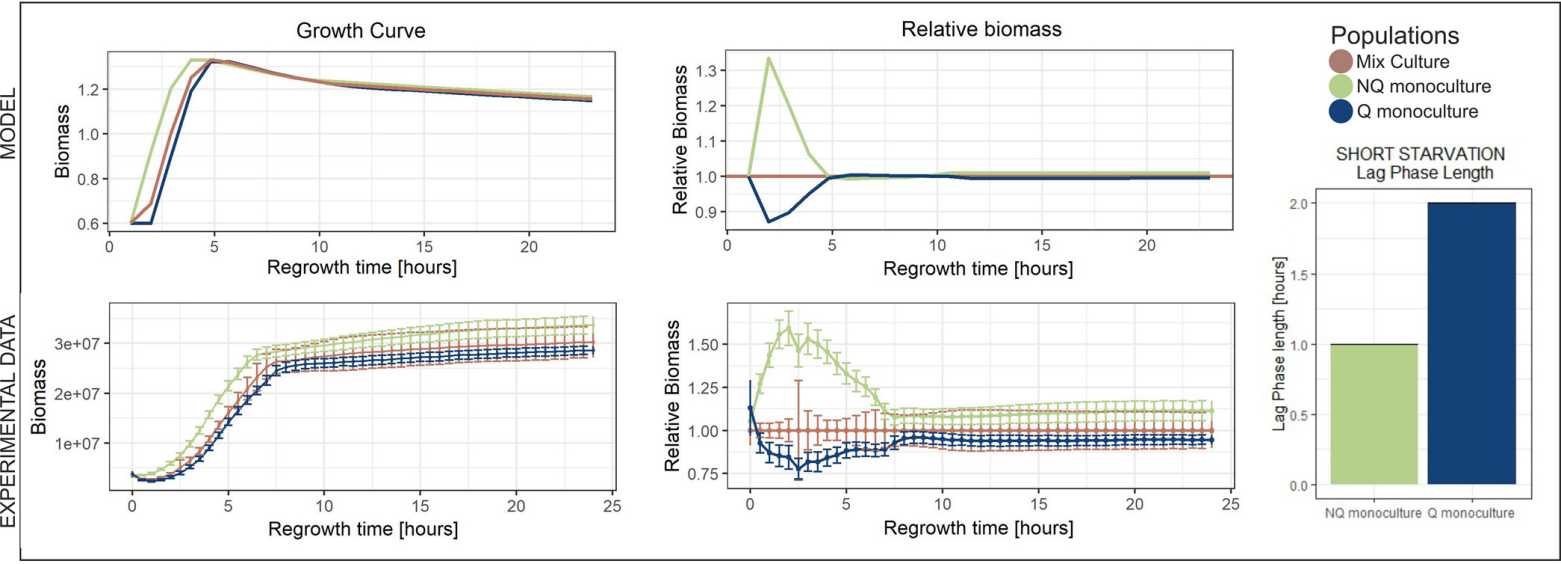
Short-starvation scenario. We modeled experimental populations in the short-starvation scenario. The NQ monoculture gained an advantage over both Q and mixed cultures up to 5 h of regrowth. The model suggested that this advantage was due to a shorter lag phase of NQ cells when starved for a short period of time. The model results were confirmed by laboratory experiment. When starved for only 4 days, NQ monoculture indeed outcompeted mixed and Q cultures at the beginning of regrowth (up to ∼7 h). Moreover, the lag phase of NQ cells was shorter than the lag phase of Q cells (the bars represent average results from 16 repetitions).

Since the disadvantage of NQ was smaller when starvation time was short, we used the short-starvation experiment to verify whether NQ cells could have an advantage over Q cells. Our model predicted that this could be the case if the lag of NQ cells was shorter than that of Q cells. To test this experimentally, we isolated Q and NQ cells from cultures that had been in the stationary phase for 4 days (short starvation). Q and NQ monocultures, as well as mixed cultures, were prepared and the cultures were placed into a fresh rich medium for regrowth. After such short starvation, the NQ monocultures restarted growth faster than the Q and mixed cultures (the average lag length for Q monoculture was 2 ± 0.0 h [mean ± standard deviation] and for NQ monoculture was 1 ± 0.0 h; *P* = 2.2 × 10^−16^). Relative-biomass analysis revealed that the advantage of NQ persisted up to 8 h after inoculation (NQ-mixed, *P* = 8.08 × 10^−4^; NQ-Q, *P* = 5.5 × 10^−7^) (Fig. 6).

## DISCUSSION

Here, we examined six types of experimental populations: three cell composition types (Q and NQ monocultures and Q-plus-NQ mixed cultures) in two starvation media (sterile water and spent medium, representing simple and complex environments, respectively). Populations were starved for 6 weeks, and the regrowth abilities (biomass increase) and lag phase length in fresh rich medium were monitored after each week. We also conducted an experiment testing the experimental populations in a very short, 4-day starvation scenario.

### Q cells are adapted to survive long starvation.

We showed that Q monocultures had a clear biomass advantage over NQ monocultures after long starvation. The difference was especially pronounced when regrowth times were short (up to 10 h), and Q monocultures’ advantage increased when populations were starved longer (Fig. 2 and 4). The biomass of Q monocultures was also higher on average than that of mixed cultures (Fig. 2 and 4), although the difference did not appear to be statistically significant (Fig. S10 and S11). It could be explained by the fact that mixed cultures were mostly composed of cells in the quiescent state. The advantage of Q over mixed cultures was also confirmed by the model (Fig. 2C and 4C). We can therefore hypothesize that it was mostly quiescent cells that were responsible for the survival and regrowth abilities of mixed cultures. This was a consequence of Q cells dying at a lower rate than NQ cells under starvation (13, 15) and having shorter lags when entering regrowth in the rich medium (15). Additionally, Q cells survived better any additional stresses, such as freezing, which preceded the long starvation (survival rates after freezing were 40% for Q and 10% for NQ cells). This, however, was not sufficient to explain that the advantage of Q over NQ cells varied with starvation and regrowth time. In fact, our model showed that freezing did not qualitatively change our results (Fig. S3). However, this advantage decreased and finally disappeared when regrowth times increased, because after a sufficiently long time for regrowth, all populations reached their carrying capacities regardless of the initial sizes and lags (Fig. S7 and S8). This was because the maximal biomass in any case was determined by nutrient abundance in a given medium. Thus, when the regrowth time was long enough, any biomass advantage would gradually decrease and finally disappear.

### Nutrient recycling increases the survival of NQ cells during long starvation in the complex environment.

We showed that while the starvation medium had little or no impact on Q monocultures and mixed cultures, the NQ monocultures survived relatively better in the complex environment (Fig. 5). Both the simple and the complex starvation media were lacking glucose; however, in the complex environment, which consisted of spent medium, nutrient and metabolite recycling was possible. This was because some nutrients, such as some amino acids, remained in the spent medium even after the onset of growth and starvation and because additional nutrients might have been released from dead cells during long starvation (28). In addition, it has been demonstrated that the excretion of nutrients and metabolites into the environment is a natural property of yeast populations and that cells can cooperatively exchange exometabolites (28–30). In the simple environment, the nutrients released from dead cells were unlikely to be reused during starvation because the environment was too poor to provide all types of nutrients necessary for growth. However, in the complex environment, released nutrients could cover missing compounds and, together with nutrients remaining in the spent medium, enable increased survival. Indeed, death and nutrient recycling has been demonstrated as potentially crucial in bacterial communities (28). We captured this phenomenon in our model by varying to what extent nutrients could be recycled. Experiments performed in complex starvation media confirmed model predictions that NQ monocultures would do better when nutrient recycling was possible (Fig. 4 and 5). This could be because the nutrient recycling simply helped to reduce the effect of death rates being higher for NQ than Q cells.

### Population dynamics during long starvation in the spent medium.

Long-term survival during stationary phase (LTSP) and the evolution of growth advantage in stationary phase (GASP) mutants are well studied in bacteria (6, 7, 31). There is also growing evidence for genetic adaptation to long-term starvation (e.g., GASP-like phenotypes) in yeast (8, 32). However, this phenomenon is understudied. In our experiments, we focused on the survival and regrowth abilities (lag phase and biomass) of populations starved for up to 6 weeks. Such periods of time could be treated as long-term starvation (32).

Given the state-of-art knowledge about the fate of Q and NQ cells during starvation (19), we made the following assumptions in our model: most Q cells will eventually become NQ cells (19); the death rate of Q cells is lower than the death rate of NQ cells (13); and Q cells do not divide, while NQ cells give rise to NQ daughters at a rate that depends on the availability of some residual nutrients in the medium (confirmed by our observations). As the turnover of nutrients is known for LTSP in bacteria, we assumed it can also happen in yeast, and, therefore, we included it in our model, where we assumed that nutrients can be reused in complex but not in simple environments. The model results confirm the potential importance of such nutrient recycling for NQ cells during long starvation in complex environments. Investigating genetic changes and evolution in LTSP is beyond the scope of the current study. Although we did not track the genetic changes in our experimental populations, we hypothesized that constant division of NQ cells in long-term starvation provides conditions for mutations and selection. As a result, in the long term, new genotypes may appear, similar to the GASP mutants appearing in bacteria (6, 31, 33). Indeed, we observed that the variability between our initially clonal replicates increased with starvation time, which supported the hypothesis that the culture experiences selection pressure. Moreover, the observed variability in growth curves and lag times was higher for NQ than for Q and mixed populations (Fig. 3 and 5, weeks 5 and 6 for NQ). This was in line with the hypothesized population dynamics, where NQ cells divide during starvation. The variability we observed might be the result of mutations arising at 3- to 6-fold-elevated rates (8) or even the appearance of mutator clones that are reported for Escherichia coli (34) and yeast (*MMS2* gene mutations) (20).

### NQ outperform Q cells when starvation is short.

Shorter starvation times resulted in smaller growth advantages of Q monocultures compared to mixed cultures (Fig. 2 and 4). This raises the question of whether there could be scenarios in which entering quiescence is not beneficial at all. The model suggested that this could be the case if the lag phase was shorter for NQ cells than for Q cells. Indeed, we showed that in the case of very short starvation (4 days), lag phase length was shorter for NQ cells (Fig. 6). In particular, we showed that when a culture faced a very short starvation period, cells that had switched to the quiescent state experienced longer lags than those that had not (Fig. 6). As a consequence, at the beginning of regrowth after short starvation, NQ monoculture gained an advantage over Q and mixed cultures.

### The presence of both Q and NQ cells could be adaptive for populations under specific ecological scenarios.

Multiple studies have demonstrated the advantages of quiescent cells over any other phenotype when populations are facing stressful conditions. Simple environments with a single, highly stressful factor (such as heat shock or toxins) indeed favor more-resistant quiescent cells (13, 15). Such tests provide important information; however, natural populations usually face fluctuating environmental changes, often with gradually increasing stress (27, 35).

There is a scarcity of data regarding the frequency of Q cells in natural populations. Most of the wild strains are not haploids and enter a sporulation program when starved. However, the Q/NQ balance in the population is evolvable. This was confirmed by selection enrichment experiments on serially propagated yeast populations that were enriched for either only Q or only NQ cell types over many repeated growth-starvation cycles. After 30 cycles (equivalent to ca. 300 generations), each enriched population produced a higher proportion of the enriched cell type than was in the starting population, which is suggestive of adaptive change. There were also observed differences in each population’s fitness, suggesting possible trade-offs: clones from NQ lines were better adapted to exponential growth, while clones from Q lines were better adapted to starvation (20).

Overall, our results show that switching to the Q state may not always be adaptive and that the benefits of this physiological transition depend on the ecological context. When the starvation period is very short (e.g., 4 days) and beneficial conditions are restored, cells that do not switch to quiescence benefit from a shorter lag phase. On the other hand, quiescent cells survive long starvation much better. Moreover, when nutrient recycling during starvation is possible, NQ cells perform as well as Q cells if the starvation is no longer than 2 weeks.

In particular, the fact that natural populations are phenotypically heterogeneous during starvation and are composed of both Q and NQ cells may not be a side effect of an imperfect switch to quiescence but may actually be a proper bet-hedging strategy under uncertain ecological conditions. It will be interesting to further test these assertions using evolution experiments in which populations are subjected to different lengths and frequencies of periods of starvation and regrowth.

## MATERIALS AND METHODS

### Strain and Q and NQ cell acquisition.

We used the laboratory haploid prototrophic Saccharomyces cerevisiae strain S288C (*MAT*α). In order to obtain Q and NQ cells, we applied a previously described fractionation procedure in a density gradient (13). In short, an overnight culture was diluted 10-fold and 100 μl (∼2× 10^7^cells) was incubated on a yeast extract-peptone-dextrose (YPD) agar plate for 4 days at 30°C (reaching a cell density of ∼2 × 10^8^/ml). The density gradient was obtained by mixing Percoll and NaCl (1.5 M) in proportions of 9:1 (vol/vol) and by subsequent centrifugation in the angular rotor for 20 min at a relative centrifugal force (RCF) of 10,078 × *g* (model MPW-352R; MPW Med. Instruments). Then, the culture was washed from the plate (10 ml 50 mM Tris, pH 7.5), and 4 ml was pelleted, placed on the top of a density gradient, and centrifuged in a swinging-bucket rotor for 60 min at an RCF of 417 × *g*. Upper (NQ cells) and lower (Q cells) fractions were carefully separated by using pipets and placed in individual tubes (Fig. 1A). For the long-starvation scenario, harvested cells were stored at −70°C in 25% glycerol until the beginning of the starvation experiment. For the short-starvation scenario, harvested cells were immediately used to prepare experimental populations and placed for regrowth.

### Experimental long-starvation scenario. (i) Preparation of experimental populations.

For the long-starvation scenario, monocultures of previously fractionated Q and NQ cells stored at −70°C were thawed. The survival rate was measured using the flow cytometry FUN LIVE/DEAD yeast viability kit according to the manufacturer’s instructions. Cells were then merged according to type (Q and NQ) and washed in sterile water 3 times. Q and NQ cells were diluted to equal densities (optical density [OD] = 0.8; all OD measurements were taken with the SpectraMax iD3 multimode microplate reader, with λ = 600 nm) in sterile water. Then, six types of experimental cultures (Q monoculture, NQ monocultures, and Q-plus-NQ mixed cultures, each in sterile water and in spent medium) were set up. Each type of experimental population was prepared in 5 repetitions, 5 ml each, giving 30 independently starved cultures altogether. Mixed cultures were set up by mixing Q and NQ cells in 3:1 (vol/vol) proportions. Mixed cultures were treated as a reference point because they mimicked the naturally occurring Q/NQ cell ratio in the S288C yeast strain. Two starvation media were used: sterile water (simple environment) and spent medium (complex environment) (Fig. 1B). To harvest spent medium, yeast cells of the same prototrophic S288C strain were inoculated into fresh YPD for 4 days, and then cells were pelleted and the supernatant was filtered and placed in a sterile container. The remaining cells were discarded. The lack of viable cells in the spent medium was confirmed by spreading samples of the harvested medium on the YPD plate and incubating for 5 days at 30°C. No colony growth was observed on these plates.

For the long-starvation scenario, the cultures were kept at 30°C with shaking for 6 weeks. Samples from starving experimental populations were checked weekly for regrowth ability (see “Regrowth procedure,” below), starting 1 week (7 days) after setting up the experimental populations.

### (ii) Regrowth procedure.

From each starving culture, a 275-μl sample was taken and spun down, supernatant (starvation medium) was discarded, and cells were resuspended in 550 μl of fresh YPD medium. Then, 200 μl was placed in a 96-well plate (flat bottom) in two repetitions. Additionally, fresh cells (inoculum; cells of the same S288C strain from liquid YPD medium incubated overnight at 30°C) were placed into the plate as a control. The plate was covered with transparent incubation foil and placed in the reader (SpectraMax iD3 multimode microplate reader) for 70 h at 30°C with shaking. OD measurements (λ = 600 nm) were taken every 30 min. The procedure was repeated weekly through the 6 weeks of the starvation experiment (Fig. 1C).

### Experimental short-starvation scenario.

Q and NQ cells were acquired in the same way as described above. Then, immediately after fractionation, Q and NQ cells were diluted to equal densities (OD = 0.4), and the mixed culture was prepared by mixing Q and NQ cells in 3:1 proportions. Q monoculture, NQ monoculture, and mixed culture were suspended in fresh liquid YPD medium. Then, 150 μl was put in a flat-bottom 96-well plate in 16 repetitions for each culture type. The plate was covered in transparent incubation foil and placed in the reader for incubation at 30°C with shaking for 24 h. OD measurements (λ = 600 nm) were taken every 30 min.

### Relative biomass analysis.

OD values were first converted into biomass (cell number) according to the following equation:

$$\text{Biomass}=-2\times 10^{6}\times {\text{OD}}^{3}+3\times 10^{7}\times {\text{OD}}^{2}+3\times 10^{6}\times \text{OD}+2.203\times 10^{5}$$

The biomass values given throughout the manuscript are the number of cells present in 200 μl, which is the total volume in a well (in 96-well plate) during regrowth. The equation was established by combining OD measurements (λ = 600 nm) and cell counting in a flow cytometer (Beckman Coulter CytoFlex) after staining with propidium iodide (PI).

The data analysis was conducted on 24 h (out of 70 h) of constant regrowth, as this is the best time frame to capture differences between experimental populations. We compared how monocultures regrew after starvation in comparison to the regrowth of mixed cultures by relative-biomass analysis. The relative biomass of a given monoculture was calculated as the ratio of its biomass and the average biomass of mixed cultures at a given time point of regrowth (weekly procedure). The relative biomass of mixed cultures equaled 1 on average (Fig. 2 and 4).

To compare the effect of the starvation medium on the experimental populations’ survival (Fig. 5), relative biomass was calculated as the ratio of the biomass of an experimental culture of a given cell type starved in the complex environment (spent medium) and the biomass of an experimental culture of the same cell type starved in the simple environment (sterile water) at a given time point.

Statistical analysis of data from several chosen time points was conducted via analysis of variance (ANOVA) as biomass of the experimental culture, followed by *post hoc* Tukey tests (Fig. S10 and S11).

### Lag phase length analysis.

Lag phase length was defined as the time needed for a population to increase its OD by 0.01 from the OD at the beginning of regrowth. Lag phase lengths of different cultures after a given week of starvation were compared using a two-sided *t* test (Table S2). In addition, we compared lag phase lengths calculated by the above-mentioned methods to those calculated as the length of time taken to reach the maximum growth rate (i.e., when the second derivative of biomass with respect to time was maximal, as explained in reference 36 and shown in Fig. S12).

### Model description.

The model simulations mirror the experimental procedures in which we first starved the cultures and then let them regrow in fresh medium. The model represents the biological reality that differentiation into Q and NQ cells starts when the nutrients are nearly depleted and that this differentiation is not instantaneous (Fig. S1A, B, and C). The model tracks the concentrations of limiting resources, nutrients available for recycling, and various types of cells over time. Population dynamics in the starvation phase is determined by the death rates of Q and NQ cells (calculated based on reference 13) and nutrient recycling (which is assumed to occur in complex but not in simple environments). The population dynamics during regrowth on fresh medium is based on the standard Michaelis-Menten kinetics, taking into account different lengths of the lag phase for Q and NQ cells.

The model is based on previously described ordinary differential equation (ODE) models that use a bottom-up approach to track the population dynamics in specific ecological contexts (28, 37, 38). The detailed description of the model and model parameters can be found in the supplemental materials. The numerical solutions of the model were obtained using MATLAB 2018a, and the parameters were fitted using the R global optimization package DEoptim (39).

### Data availability.

Experimental data generated in the laboratory during this study have been deposited in GitHub (https://github.com/bognabognabogna/Q-NQ-data-analysis/tree/master/data). The model code is publicly available on GitHub (https://github.com/bognabognabogna/Q-NQ-data-analysis/tree/master/Matlab_scripts).
