# Supplementary material for: On the Ecological Significance of Phenotypic Heterogeneity in Microbial Populations Undergoing Starvation
Source: Microbiol Spectr. 2022 Jan 12;10(1):e00450-21. doi: 10.1128/spectrum.00450-21 (PMC8754142; doi:10.1128/spectrum.00450-21)
Supplement: SUPPLEMENTAL FILE 1 — Supplemental material. Download SPECTRUM00450-21_Supp_1_seq1.pdf, PDF file, 4.0 MB [file spectrum00450-21_supp_1_seq1.pdf]

Supplementary Tables and Figures

Environment	Cell type	WEEK	LAG PHASE LENGTH
Simpe Environment	NQ	1	2.20 ± 0.45 h
		2	4.10 ± 0.22 h
		3	5.00 ± 2.09 h
		4	7.50 ± 4.43 h
		5	7.63 ± 6.41 h
		6	9.40 ± 4.99 h
	Q	1	1.50 ± 0.00 h
		2	1.80 ± 0.27 h
		3	1.70 ± 0.27 h
		4	2.80 ± 0.57 h
		5	3.30 ± 1.30 h
		6	1.60 ± 0.89 h

Environment	Cell type	WEEK	LAG PHASE LENGTH
Complex Environment	NQ	1	2.00 ± 0.00 h
		2	2.10 ± 0.42 h
		3	2.38 ± 0.25 h
		4	4.00 ± 0.61 h
		5	7.20 ± 5.39 h
		6	6.25 ± 2.47 h
	Q	1	1.60 ± 0.42 h
		2	2.10 ± 0.22 h
		3	1.50 ± 0.00 h
		4	2.80 ± 0.27 h
		5	2.80 ± 0.27 h
		6	2.60 ± 1.47 h

Tab. S1 – Lag phase length of Q and NQ monocultures during long starvation experiment, starved in simple (sterile water) or complex environment (spent medium)

Results from statistical analysis:

```
lag ~ week * starvation_medium * cell_type

Residuals:
    Min      1Q  Median      3Q     Max 
-6.6022 -0.6364  0.0352  0.6273 10.6859 

Coefficients:
                                          Estimate Std. Error t value Pr(>|t|)    
(Intercept)                                 1.0506     0.9134   1.150 0.252652    
week                                        1.4103     0.2372   5.945 3.58e-08 ***
starvation_medium_used YPD                   -0.9611     1.3303  -0.723 0.471578    
cell_typeQ                                  0.4561     1.2904   0.353 0.724464    
week:starvation_medium_used YPD              -0.2654     0.3581  -0.741 0.460203    
week:cell_typeQ                            -1.2360     0.3332  -3.709 0.000333 ***
starvation_medium_used YPD:cell_typeQ        0.8817     1.8561   0.475 0.635738    
week:starvation_medium_used YPD:cell_typeQ   0.3275     0.4878   0.671 0.503433    
---
Significance codes:  0 ‘***’ 0.001 ‘**’ 0.01 ‘*’ 0.05 ‘.’ 0.1 ‘ ’ 1

Residual standard error: 2.189 on 106 degrees of freedom
Multiple R-squared:  0.5141,     Adjusted R-squared:  0.482 
F-statistic: 16.02 on 7 and 106 DF,  p-value: 3.221e-14
```

Tab. S2. We verified the effect of starvation length ('week'), culture type ('cell type'), and starvation medium ('starvation_medium ') on the lag phase using a linear model. We report the p-values from F-tests on regression coefficients.

Fig. S1A

Scheme of cell differentiation we represent in the model. The figure is based on Allen et.al 2006

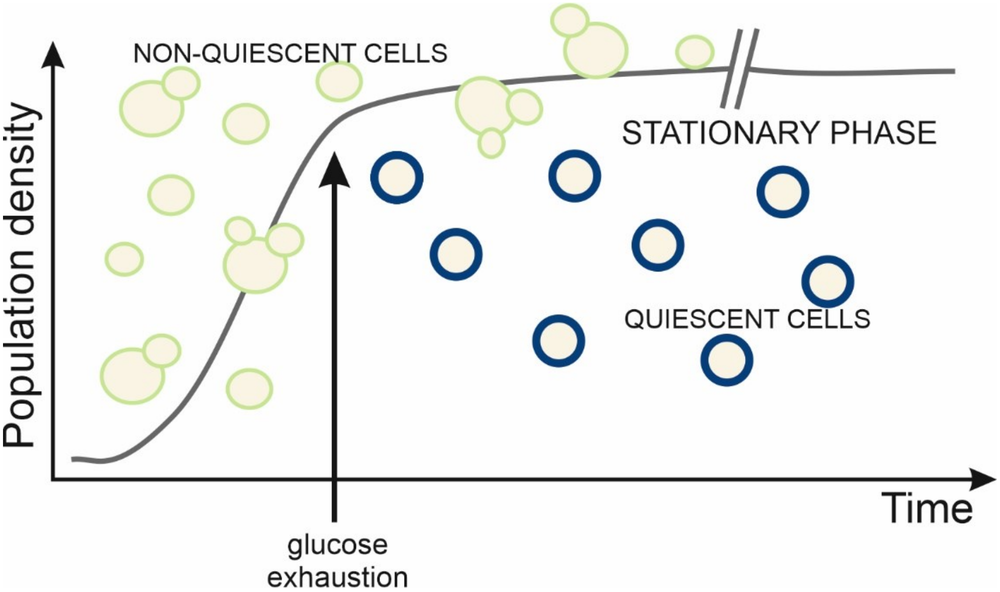

The process of becoming a Q cell is not instantaneous. In order to start this process, NQ cells need to have some energy in the system and the proper Q cells appear after a couple of days of starvation i.e. when glucose has already been depleted. The intermediate variable *NQprep* in the model represents that behavior and can be understood as the cells that are in the process of switching to Q.

Fig. S1B

Modeled dynamics of Q (navy) / NQ (green) cell balance within 4 days of culture's growth.

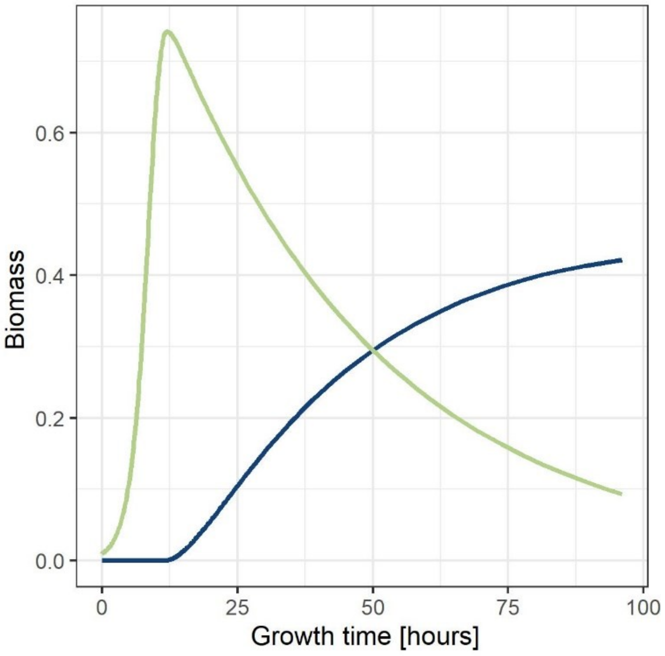

Fig. S1C. Modeled dynamics of switch rate to quiescence state, which depends on available glucose concentration.

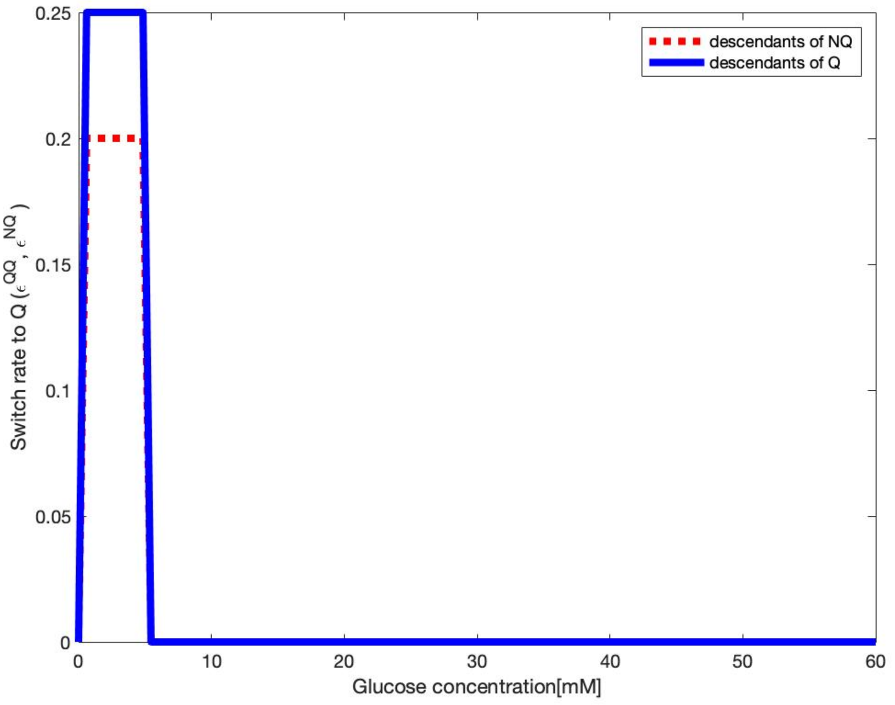

Fig. S2. Fitting model (predicted) to experimental data (real).

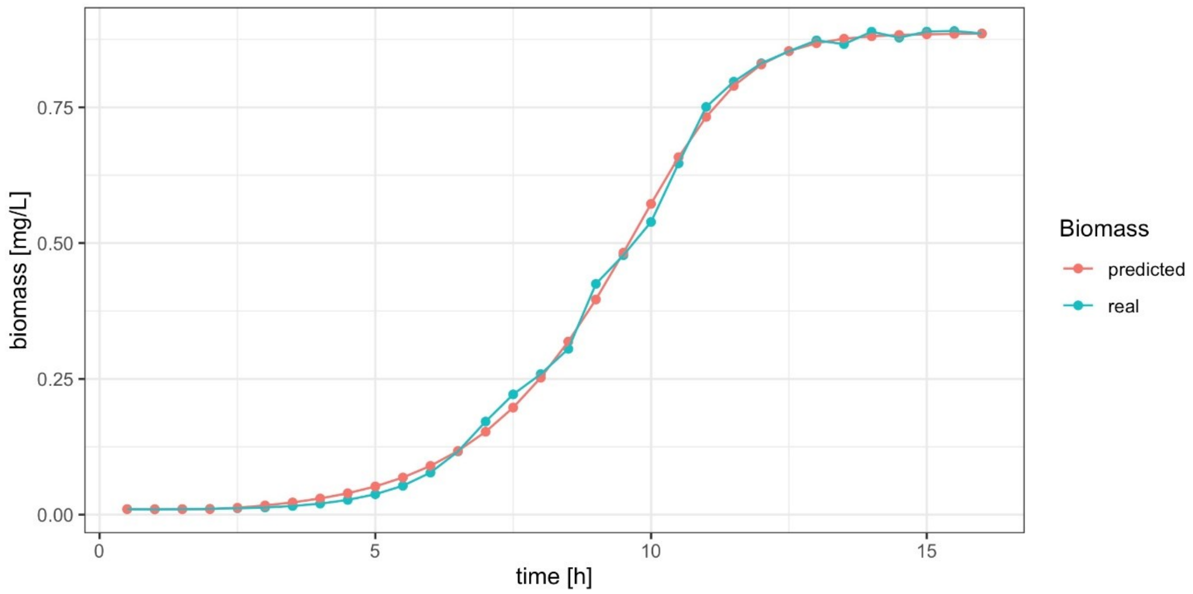

Fig. S3. The survival rate connected with freezing is 0.4 and 0.9 for Q and NQ cells respectively. The model shows that the effect of freezing does not change the data qualitatively (panel B and C).

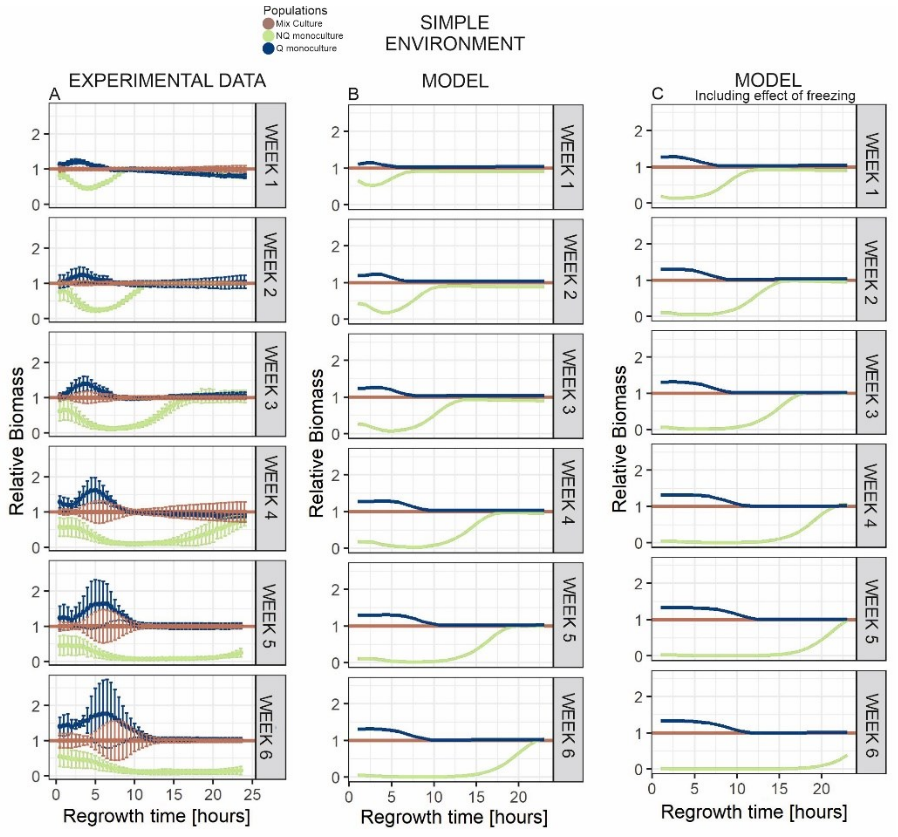

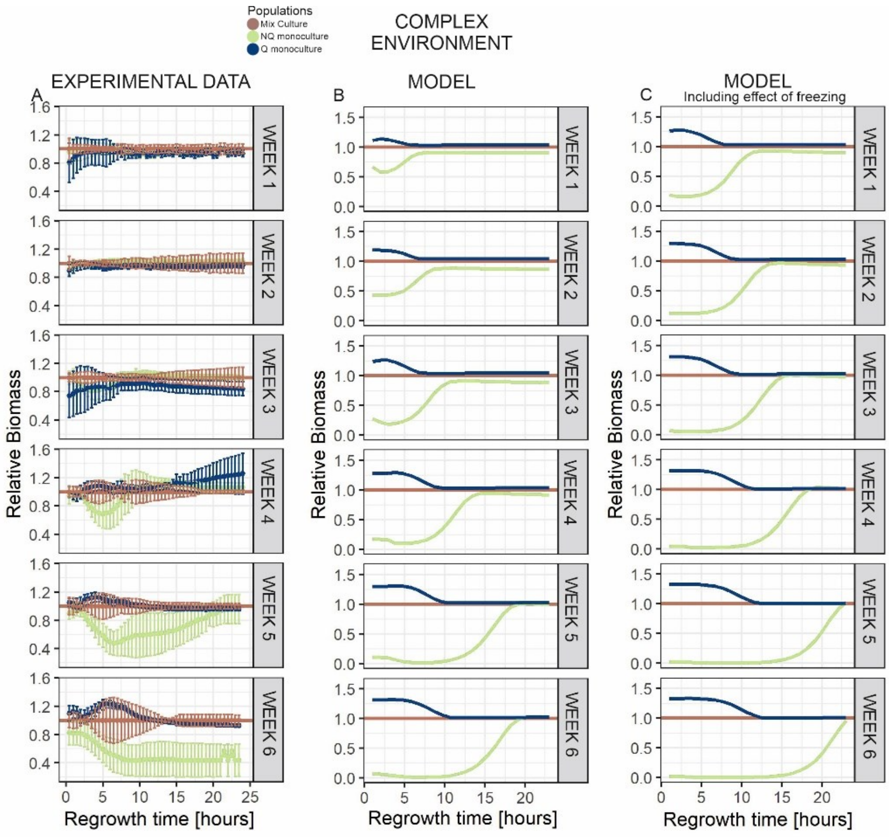

Fig. S4. Comparison of model results with alternative death rates during starvation in the simple environment. Relative biomass for results obtained from experiments are shown in the first column. The middle column shows modeled results with a death rate calculated based on Allen *et al.* 2006, which yields the best quantitative fit to experimental results. However, model results do not change qualitatively if different death rates are used (third column, calculated based on Lee *et al.* 2016). The regrowth time is shown in hours.

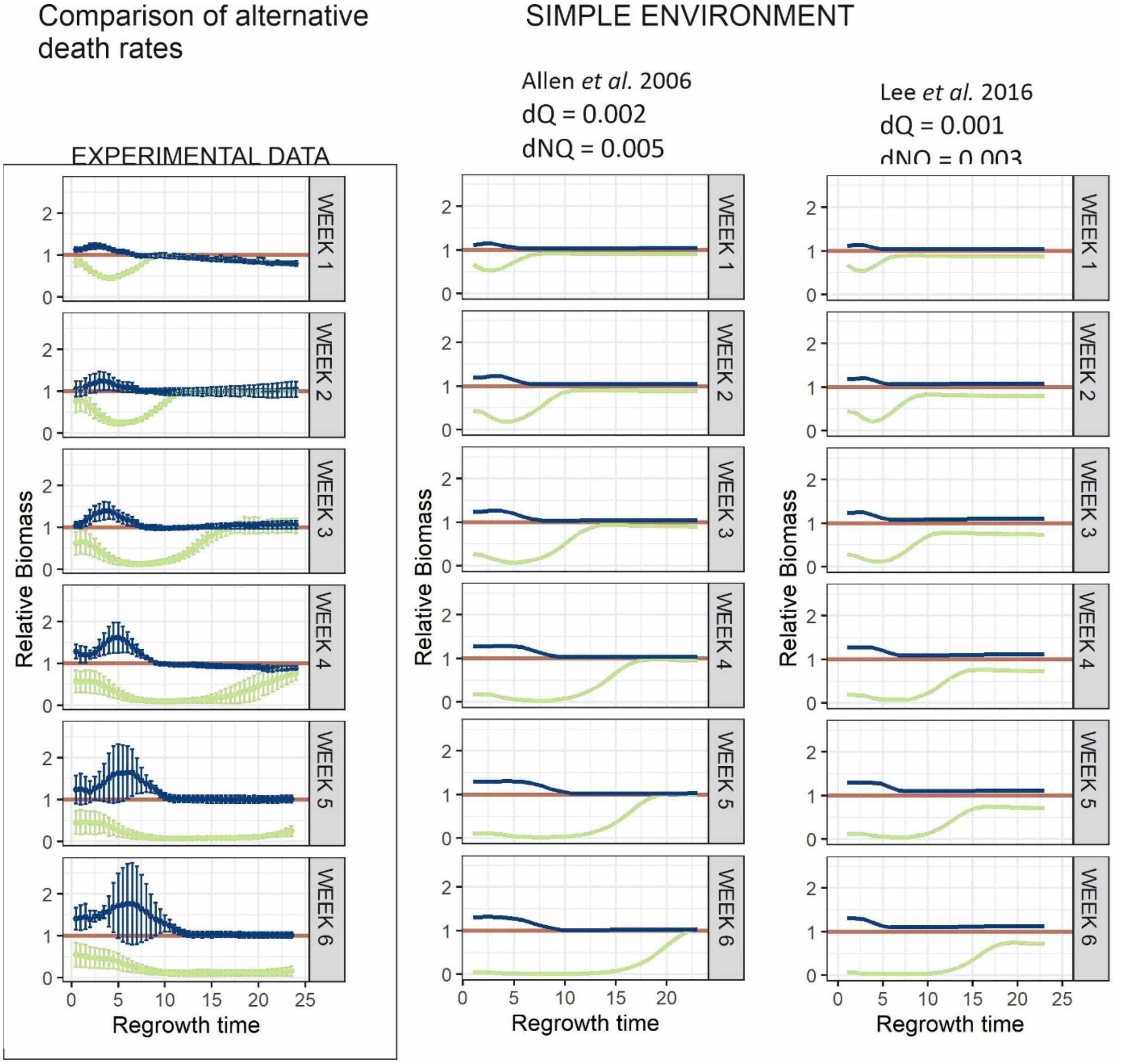

Fig. S5. Comparison of model results with alternative death rate for starvation in the simple environment. Relative biomass for results obtained from experiments are shown in the first column. The middle column shows modeled results with a death rate calculated based on Allen *et al.* 2006, which yields the best quantitative fit to experimental results. However, model results do not change qualitatively if different death rates are used (third column, calculated based on Lee *et al.* 2016). The regrowth time is shown in hours.

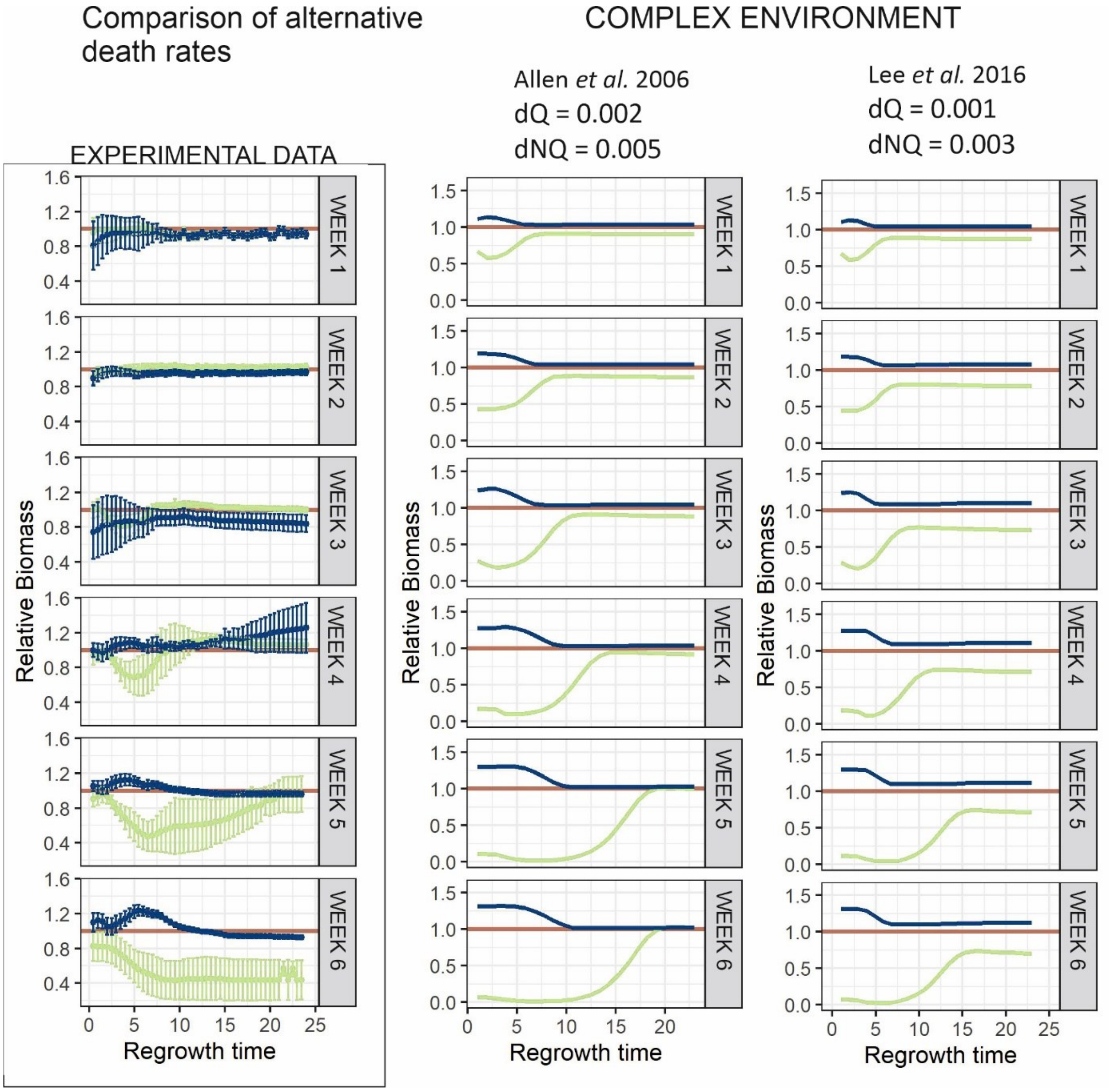

Fig. S6. Results from a simplified model where:

A. both types of cells have death rate equal to zero and Q have a lag phase constant with respect to starvation time, and shorter than NQ. The dependence of the relative biomass on the regrowth time is similar to the one obtained from the main model
B. both types of cells have no lags, and they only differ in the death rate, which is assumed to be higher for NQ than for Q. The dependence of the relative biomass on the starvation time is preserved
C. both types have death rate equal to 0, and they only differ in lag time, which is assumed to be constant for Q and consistently growing for NQ with starvation time time). The dependence of the relative biomass on the starvation time is preserved
D. both Q and NQ cells have no lags. Relative biomass of NQ cells compared in complex to simple environment

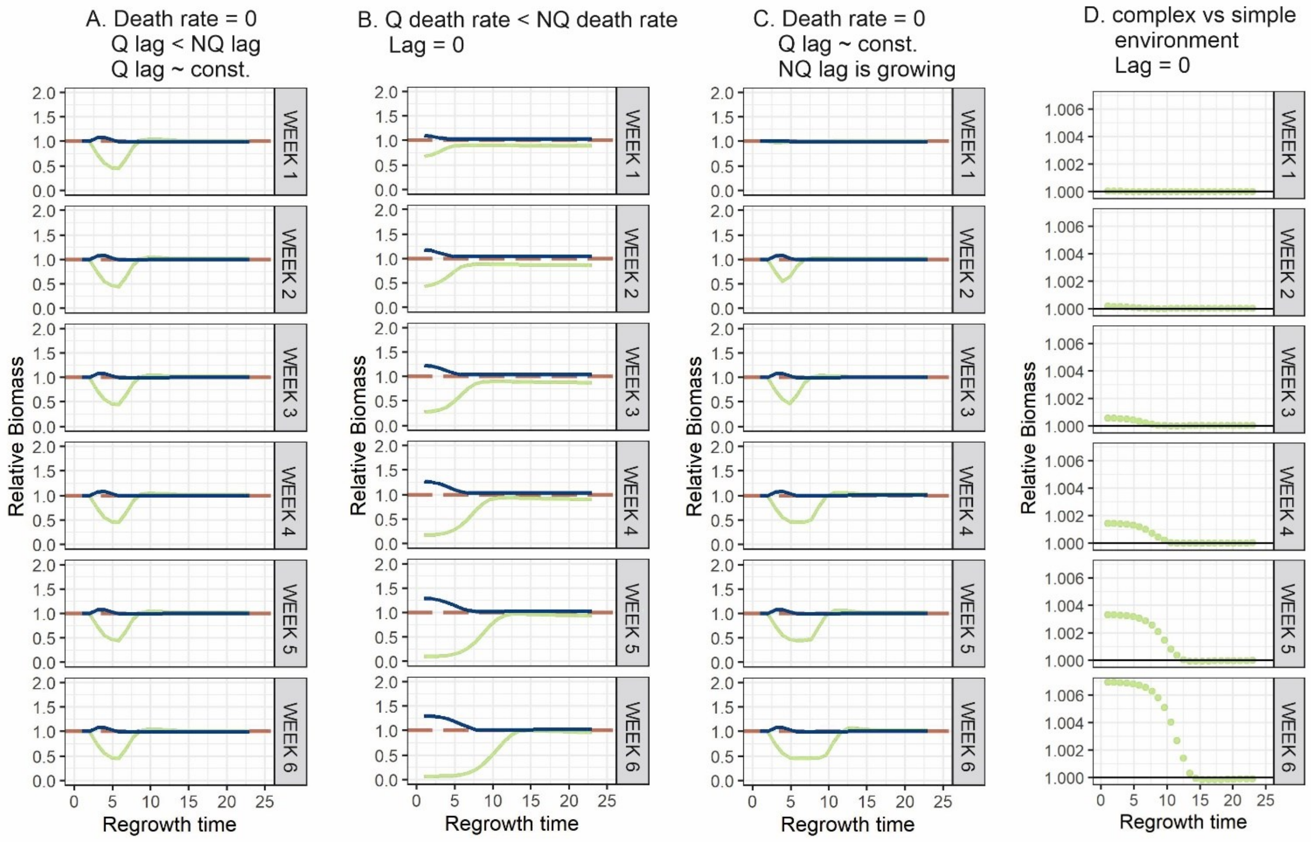

Fig. S7. Growth curves of experimental cultures starved in the simple environment, data for 70 hours of measurements.

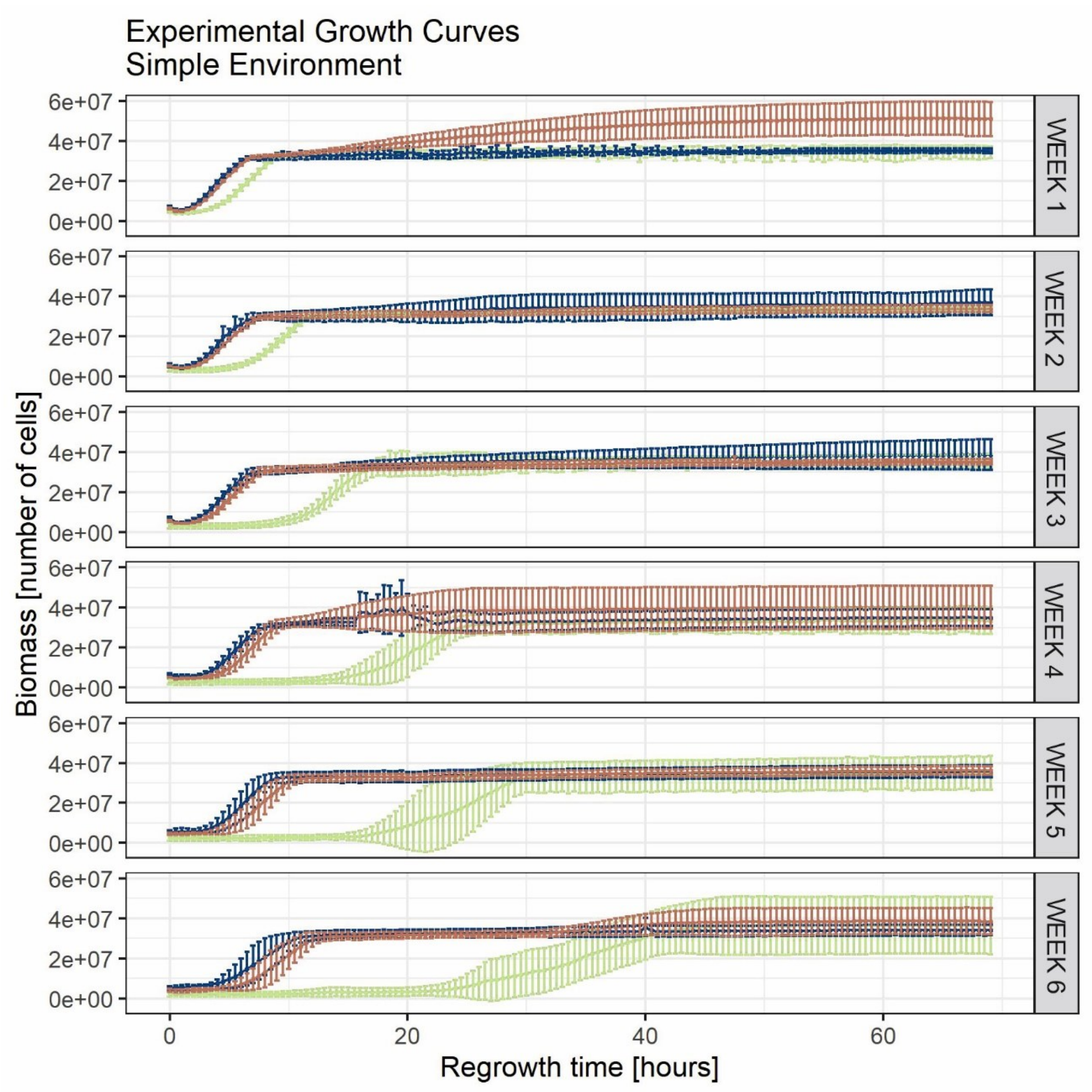

Fig. S8. Growth curves of experimental cultures starved in the complex environment, data for 70 hours of measurements.

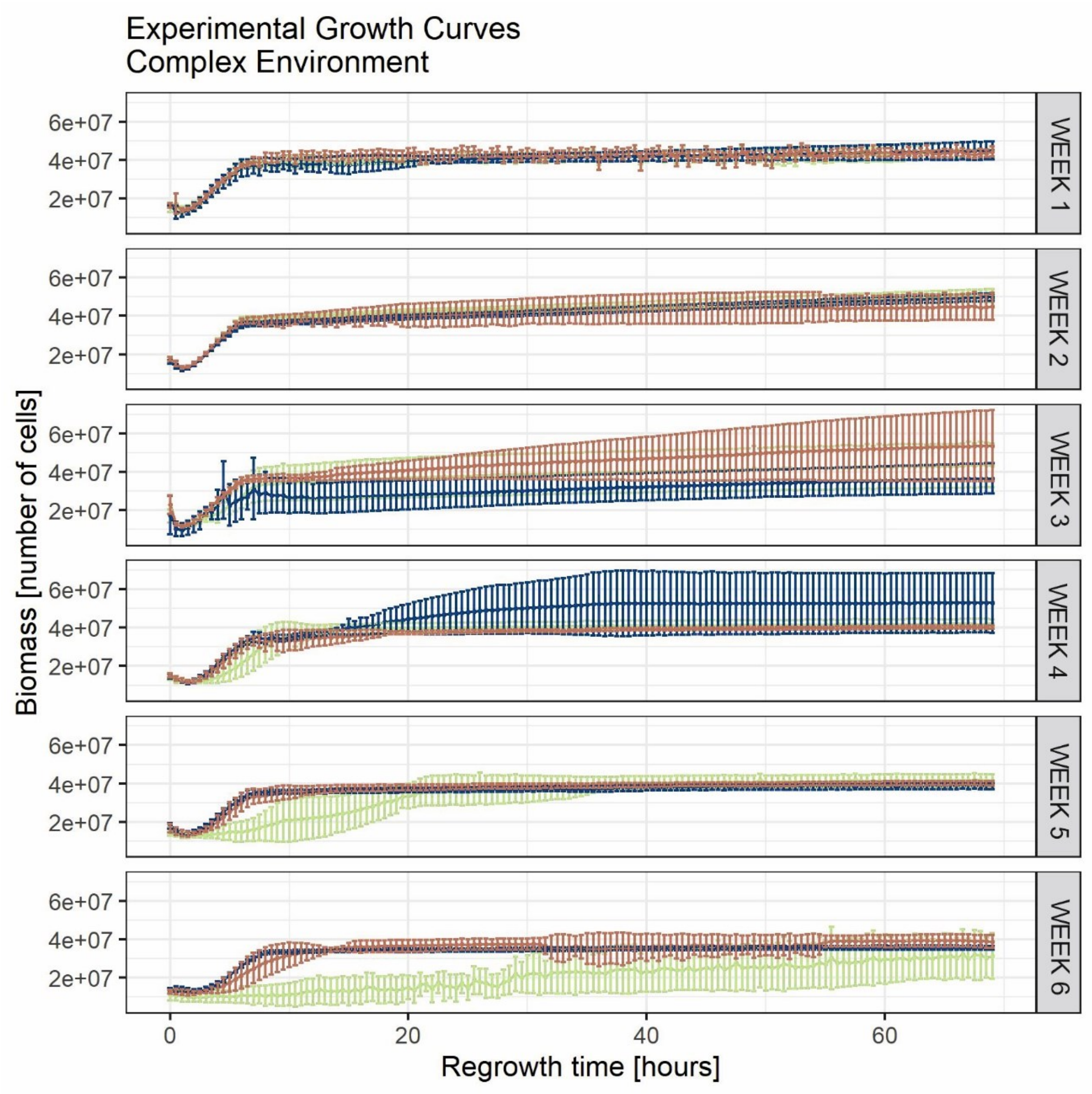

Fig. S9. – Length of lag phase for mix cultures during long starvation experiment.

The length of the lag phase is the time needed for a cell to restart the mitotic cell cycle. However, in our experiments we use a population level approach to measure lag phase length based on OD measurements – in particular we define lag phase as the time needed for a population to increase its biomass by 0.01 from the initial OD value (at the beginning of regrowth).

Mix cultures were prepared by mixing Q and NQ cells in proportions 3:1 v/v. As the population itself is heterogenous, the cells are expected to restart divisions at varying times, depending on whether a given cell is or is not in the quiescent state. As a consequence, lag phase length for mix cultures is an averaged lag of Q and NQ cells that make up the culture. Taking this into account, we decided to exclude mix cultures from the main analysis of a lag phase length (described in this publication).

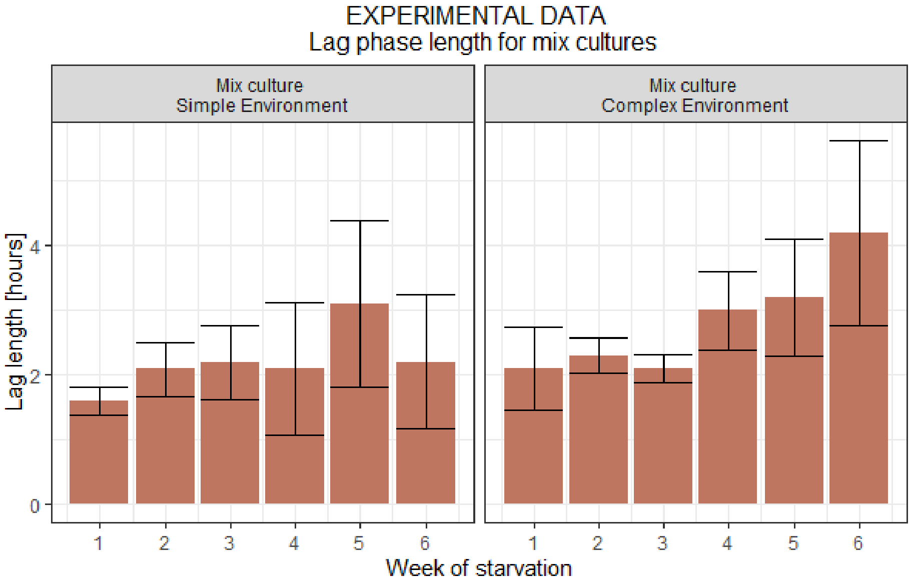

Fig. S10. Simple environment - column plots for statistically analyzed timepoints. The presented biomass values are numbers of cells present in 200µl, which is the total volume of regrowth medium. The statistically significant differences between populations are marked with asterisks.

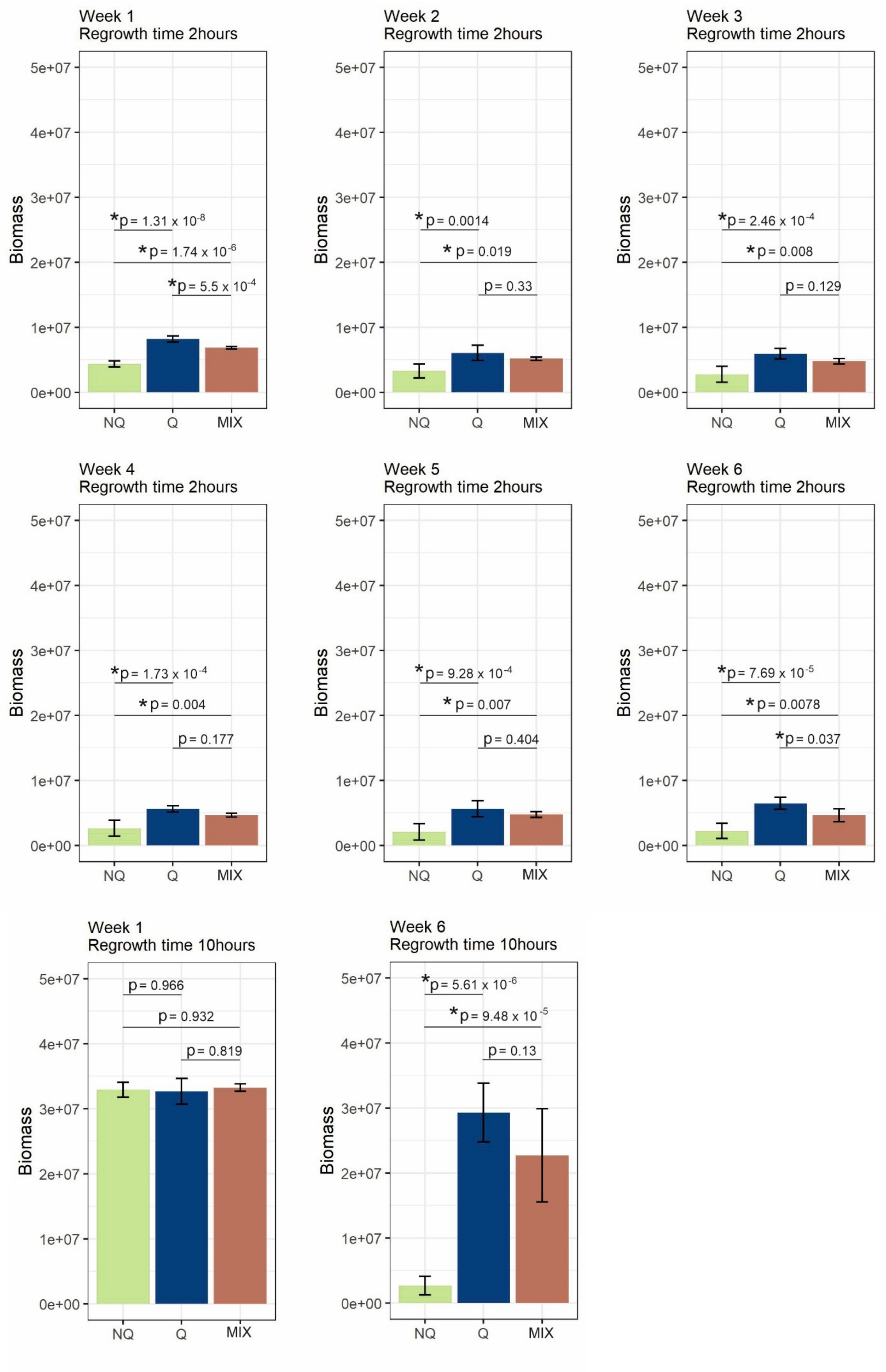

Fig. S11. Complex environment - column plots for statistically analyzed timepoints. The presented biomass values are numbers of cells present in 200µl, which is the total volume of regrowth medium. The statistically significant differences between populations are marked with asterisks.

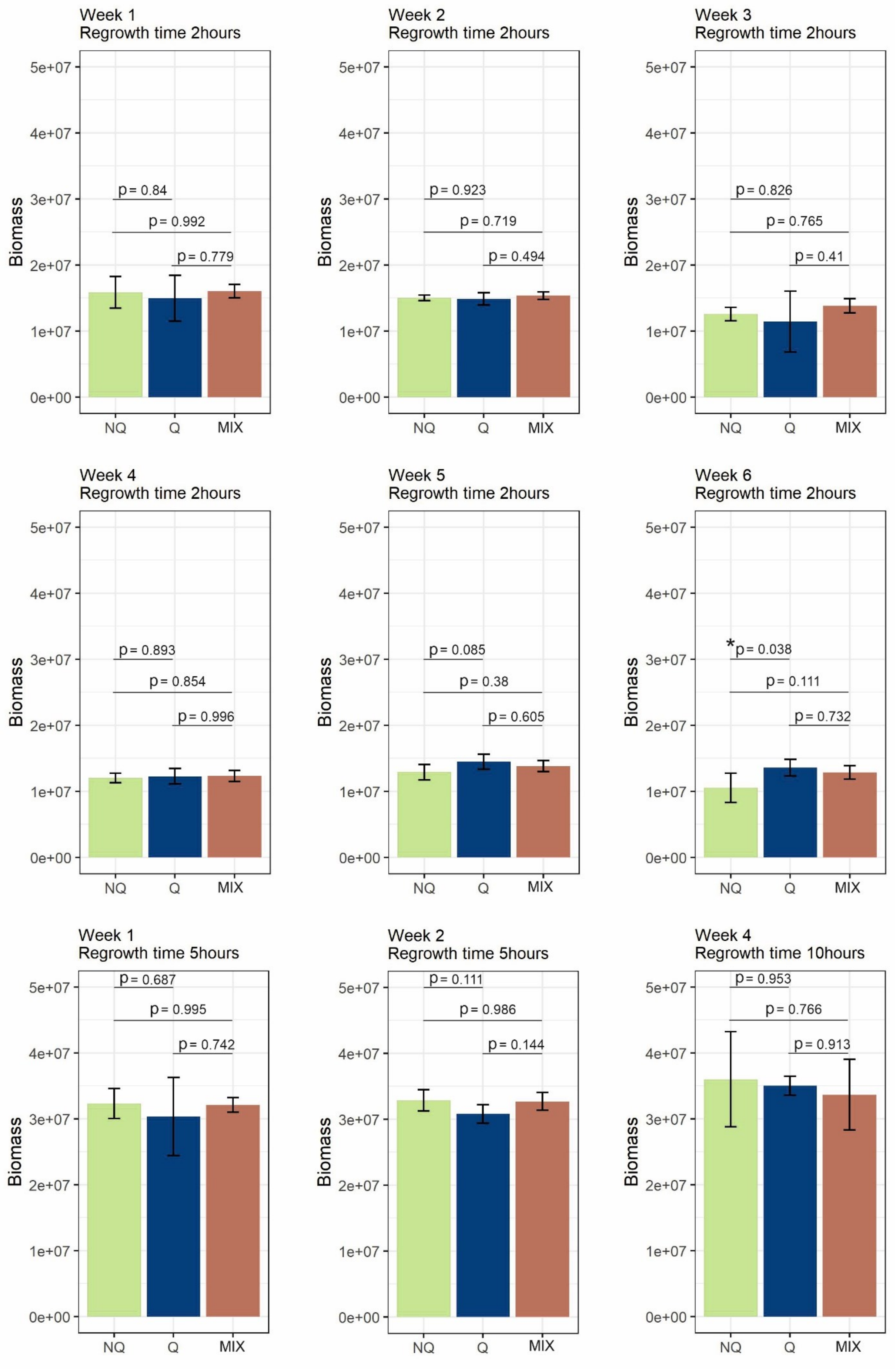

Fig. S12. **Calculation of lag phase length**

We compared lag phase lengths calculated by us to the those calculated as the length of time taken to reach the maximum growth rate (or, in other words, the time when the second derivative of biomass with respect to time is maximal, as explained in (Bertrand, 2019)).

The times taken to reach the maximum growth rate (Fig. S12) are similar to lags shown in Fig 5. In particular, they are much higher for NQ than for Q, and they tend to increase with time spent in starvation.

However, they may not represent the true biological lags. This is because the growth curves after long starvation (especially after 5 or 6 weeks) are flat and there is a lot of noise during their lag phases. As a consequence, the maximal growth rates are often incorrectly assigned to some small stochastic increases in biomass during the lag phase

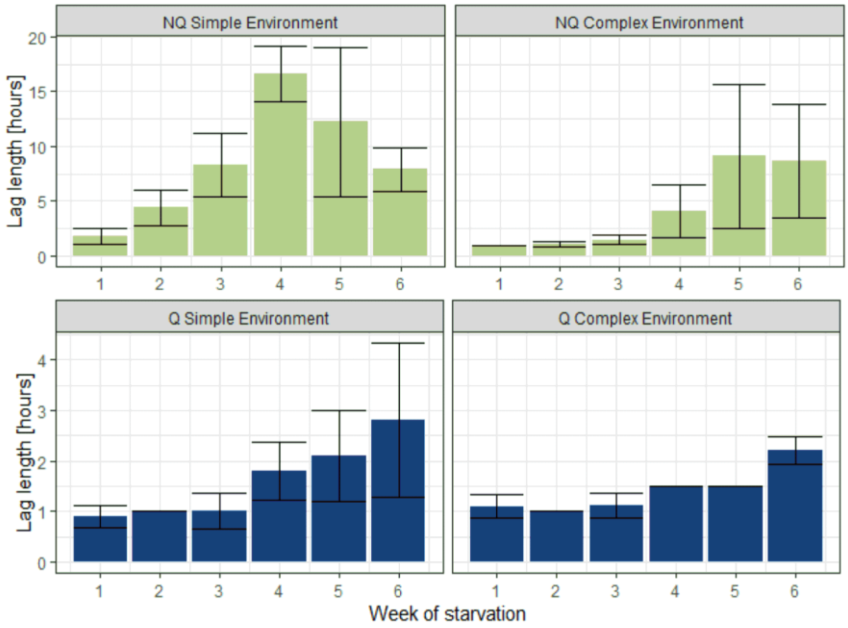

Fig. S12. The average (±SD) taken form experimental populations to reach maximum growth rate

Mathematical Model: description

In order to describe the full dynamics of a non-homogeneous population of cells under growth and starvation, we track the following variables in time:
N - concentration of non-quiescent cells that are not in the process of switching to the Q state
Q - concentration of quiescent cells
N_{prep} - concentration of non-quiescent cells that are accumulating resources and preparing to switch to Q
G – concentration of glucose (or other limiting carbon source)
A – concentration of amino acids and other substrates released from dead cells

Moreover, we divide the non-quiescent cells into:
N_Q – concentration of N-cells whose ancestors were quiescent under starvation
N_N - concentration of other N-cells, i.e. fresh cells and those whose ancestors were also non-quiescent under starvation. The N_Q and N_N concentrations are assumed to have the same growth and death dynamics. However, they differ at the very beginning and the very end of the growth phase because descendants of quiescent cells start growing after a different time lag than descendants of non-quiescent cells, and descendants of quiescent cells are more likely to switch back to Q than descendants of non-quiescent cells when there is little glucose in the environment.

(i) Growth dynamics

Our model of the growth phase is based on metabolic models of *S. cerevisiae* proposed in MacLean 2010, Lindsay 2018, Lindsay 2019, in which glucose uptake *J(G)* for non-quiescent cells has Michaelis-Menten form:

$$J(G) = \frac{VG}{K+G},$$

where V denotes the maximal rate of glucose uptake pathway, and *K* denotes the Michaelis-Menten constant (so that if *K=G, J(G)* = *V*/2).

Further, if cells are exposed to a new environmental condition (e.g. if they are supplied with glucose after a period of starvation) they are expected to start growing after some time lag,. t_{lag}^{N} for *N*-cells, and t_{lag}^{Q} for *Q*-cells. Then the uptake rate can be expressed as:

$$J^{t_{lag}} = \begin{cases} J(G)\ if\ \ t > t_{lag} \\ \mathbf{0} \qquad if\ t \le t_{lag} \end{cases}$$

(where t_{lag} is either $t_{lag}^{N}\ or\ t_{lag}^{Q}$). The efficiency of converting glucose into biomass, is described by a parameter n_e^G , which for simplicity is assumed to be constant.

(ii) Death rate

All cells are subject to a death rate, which for simplicity is assumed to be constant over time. We assume that quiescent cells are more robust to stress and therefore that their death rate d_Q is lower than the death rate of non- quiescent cells, d_N. If there are available substrates, the death rate will be compensated by growth and therefore the population will grow. Conversely, if no growth is possible the population will decline. Thus, the death rate will govern the dynamics in starvation.

(iii) Differentiation into Q and N

When there is little glucose in the environment, *non*-quiescent cells will start differentiating into quiescent cells. Because this process is not instantaneous, we track the concentration of cells that

are at some intermediate state, N_{prep}. Thus, we assume non-quiescent cells transition into the intermediate state N_{prep} with some glucose dependent rate, and the N_{prep} cells subsequently become Q by transitioning into Q with a constant rate $\epsilon,^{Q}$.

The rate of transition into N_{prep} depends on glucose in such a way that it is higher than zero only for some small but non-zero concentrations of glucose (Fig S1C). Indeed, if the glucose concentration is high there is no signal to switch to Q. Moreover, because transitioning into Q requires some energy, it is impossible to switch when there when the glucose concentration is very low.

The rate of transition into N_{prep} is also lower for descendants of non-quiescent cells than for descendants of quiescent cells: $\epsilon^{NQ}(G)$ < $\epsilon^{QQ}(G)$. This is a simplified way of describing the known phenomenon that daughters of quiescent cells are more likely to become Q cells in the next iteration of starvation than daughters of non-quiescent cells.

Note that, in our model, we assume further descendants (eg granddaughters) of quiescent cells are also more likely to become Q cells, which may not be a perfect representation of reality. However, we believe it is a reasonable simplification of the fact that cells 'remember' their state. We will further investigate this assumption.

In addition, the transition to Q requires some energy. Thus, if a cell accumulates enough energy to divide in the near future, but instead it becomes quiescent, we see it as a loss of energy from the system. Therefore, we introduce a parameter that describes the efficiency of becoming Q, $\eta^{Q} \in [0,1]$, which is typically < 1.

(iv) Resource recycling

After long enough starvation, dead cells will release amino acids into the environment, which can further decompose and provide some carbon source for the starving cells (substituting glucose). The rate of decomposition into simple carbon source sis described by a constant ϵ^{AG}. We also note that not all biomass will ever form the new carbon source, and the proportion of dead biomass that can be recycled is described by the parameter $r \in [0,1]$.

(v) Full model

We assume that the environment is well-mixed and that the studied concentrations of glucose (G), aminoacids (A), and the densities of cells do not depend on their spatial location. Then the above assumptions (i)-(iv) generate the following Ordinary Differential Equation (ODE) model describing the dynamics of a single batch culture where fresh cells are either supplied with a resource (G>0, $N_N > 0, N_Q = 0, N_{prep}$=0, $Q = 0, A = 0$) or where cells differentiated into quiescent and non-quiescent are exposed to starvation (G=0, $Q > 0$) as in our experiments.

$$\frac{\mathrm{dG}}{\mathrm{dt}} = -J^{t^{Q}_{lag}}\mathrm{N_Q} - J^{t^{N}_{lag}}\mathrm{N_N} + \epsilon^{\mathrm{AG}}\mathrm{A}$$

$$\frac{dN_Q}{dt} = n_e^G J^{t^{Q}_{lag}}\mathrm{N_Q} - d_N N_Q - \epsilon^{QQ} N_Q$$

$$\frac{dN_N}{dt} = n_e^G J^{t^{N}_{lag}}\mathrm{N_N} - d_N N_N - \epsilon^{NQ} N_N$$

$$\frac{dN_{prep}}{dt} = -d_N N_{prep} + \eta^{Q}(\epsilon^{NQ} N_N + \epsilon^{QQ} N_Q) - \epsilon^{Q} N_{prep}$$

$$\frac{dQ}{dt} = \epsilon^{Q} N_{prep} - d_Q Q$$

$$\frac{dA}{dt} = r\left(d_N\left(N_Q + N_N + N_{prep}\right) + d_Q Q\right) - \epsilon^{AG} A$$

(vi) Some simplified cases:

The model described above can be run with many possible initial conditions and parameters, depending on the experimental setup one wants to reproduce. Here we illustrate some specific, simplified scenarios, which reproduce some basic population dynamic scenarios.

a) Starvation in water:

If a culture is started with freshly obtained quiescent and non-quiescent cells ($N_N(0) > 0$, $Q(0) > 0$, $N_Q(0) = 0$, $N_{prep}(0) = 0$), and in the absence of any nutrients, we can assume: G(0)=0 (no initial glucose). Moreover, we can neglect the nutrients recycling effect as the culture is unlikely to contain all resources needed for growth even if some simple carbon source will be created. Thus, we set r=0 and we get G=0 and $\epsilon^{NQ} = 0$ for the duration of starvation. Then the model tracking the cells simplifies to:

$$\frac{dN_N}{dt} = -d_N N_N$$

$$\frac{dQ}{dt} = -d_Q Q$$

b) Short growth phase:

If a culture is started with fresh cells in a new batch culture $(G(0) > 0, N_N(0) > 0, Q(0) = 0, N_Q(0) = 0, N_{prep}(0) = 0, A(0) = 0)$ and grown for a short period of time (short enough so that glucose is not depleted), we can assume G > 0 for the duration of growth and therefore: $\epsilon^{NQ} = \epsilon^{QQ}$ = 0. Additionally, on a short timescale the nutrient recycling effect will be negligible as A~0, and we get:

$$\frac{\mathrm{dG}}{\mathrm{dt}} = -J^{t_{lag}^N}\mathrm{N_N}$$

$$\frac{dN_N}{dt} = nJ^{t_{lag}^N}\mathrm{N_N} - d_N N_N$$

c) Short regrowth:

If quiescent and non-quiescent cells are allowed for regrowth from some final starvation densities, Q_{final} *and* N_{final} , then we assume: Q(0)=0, $N_N(0) = N_{final}, N_Q(0) = Q_{final}$. Additionally, in a short time span G > 0 and therefore: $\epsilon^{NQ} = \epsilon^{QQ}$ = 0. Additionally A~0 and so have:

$$\frac{\mathrm{dG}}{\mathrm{dt}} = -J^{t_{lag}^Q}\mathrm{N_Q} - J^{t_{lag}^N}\mathrm{N_N}$$

$$\frac{dN_Q}{dt} = nJ^{t_{lag}^Q}\mathrm{N_Q} - d_N N_Q$$

$$\frac{dN_N}{dt} = nJ^{t_{lag}^N}\mathrm{N_N} - d_N N_N$$

In this scenario the only difference between descendants of quiescent and non-quiescent cells is the lag they experience before regrowing.

Mathematical model: simulations

Using our model we simulate a number of scenarios:

a) To simulate the part of our experiment illustrated in Fig 1A that consists of the process of a batch growth, stationary phase and differentiation of cells into Q we run our model (described in (v) and simplified in (vi)b) with the following initial conditions: $\boldsymbol{G(0)} = \boldsymbol{G_0},\ N_Q(0) = 0,\ \boldsymbol{N_N(0)} = \boldsymbol{P_0},\ N_{prep}(0) = 0,\ Q(0) = 0,\ A(0) = 0.$

Where:
$\boldsymbol{G_0} = \boldsymbol{111\ mmol/L}$ **and**
$\boldsymbol{P_0}$ =0.01 mg protein/L equivalent to 33*10^4 cells/L
which represents the experimental values.

The simulation is run for experimental time of 96 hours and all parameters are set to default (Table S2).

b) To simulate the part of our experiment illustrated in Fig 1B that consists of the long term starvation in H20 we run our model (described in (v) and simplified in (vi)a) with the following initial conditions:

$$\boldsymbol{G(0)} = \boldsymbol{0},\ N_Q(0) = 0,\ \boldsymbol{N_N(0)} = \boldsymbol{(1-f)P_0},\ N_{prep}(0) = 0,\ \boldsymbol{Q(0)} = \boldsymbol{fP_0},\ A(0) = 0.$$

Where:

$\boldsymbol{P_0}$ =0.6061 mg protein/L equivalent to 2*10^7 cells/L
and $f = 0$ for the NQ monoculture, $f = 0.75$ for the S culture, and $f = 1$ for the Q monoculture which represents the experimental values.

The simulation is run for the experimental time of starvation **T** (eg 4 weeks), r=0, and all the other parameters are set to default (Table S2). The concentrations after starvation are denoted by:

$$G_T = G(T),\ N_{QT} = N_Q(T),\ N_{NT} = N_N(T),\ N_{prepT} = N_{prep}(T),\ Q_T = Q(T),\ AT = A(T).$$

(c) To simulate the part of our experiment illustrated in Fig 1C that consists of the regrowth after starvation we run our model (described in (v) and simplified in (vi)c) for the experimental regrowth time of (eg 24 hours) and with the following initial conditions:

$$G(0) = G_0,\ N_Q(0) = Q_T\ \ N_N(0) = N_{NT} + N_{prepT} + N_{QT},\ N_{prep}(0) = 0,\ Q(0) = 0,\ A(0) = 0.$$

Here $G_0 = 111\ mmol/L$ which represents the experimental value.

d) Note that the long term starvation in H20 and the subsequent regrowth on fresh medium as done in our experiments (Fig 2) can be simulated by running the model as described in (b) and a subsequent run as described in (c).

Furthermore, to simulate the long term starvation in YPD and the subsequent regrowth on fresh medium as also done in our experiments (Fig 3) we run the model as in the H20 scenario, but we assume r=0, which implies that the nutrients coming from dead cells cannot be recycled during starvation.

e) To simulate the short starvation scenario (Fig 6) that consists of immediate regrowth after a short starvation we run our model (described in (v) and simplified in (vi)c) for the experimental regrowth time of (eg 24 hours) and with the following initial conditions:

$G(0) = G_0,\ N_Q(0) = Q_T\ \ N_N(0) = N_{NT} + N_{prepT} + N_{QT},\ \ N_{prep}(0) = 0,\ \ Q(0) = 0,$
$A(0) = 0.$

Here $G_0 = 111\ mmol/L$ which represents the experimental value.

$\boldsymbol{G(0)} = G_0,\ N_Q(0) = \boldsymbol{fP_0},\ \boldsymbol{N_N(0)} = (\mathbf{1} - \boldsymbol{f})\boldsymbol{P_0},\ N_{prep}(0) = 0,\ \boldsymbol{Q(0)} = \mathbf{0},\ A(0) = 0.$

Here $G_0 = 111\ mmol/L$,$\boldsymbol{P_0}$ =0.6061 mg protein/L equivalent to 2*10^7 cells/L, and $f = 0$ for the NQ monoculture, $f = 0.75$ for the S culture, and $f =$ 1 for the Q monoculture which represents the experimental values.

Mathematical Model: Default Parameters

Throughout the study we assume that 1 mg of biomass is equivalent to 33*10^6 yeast cells, as proposed in MacLean et al. 2010: V, K, n_e^G related to microbial growth were fitted from growth curves of fresh cells using the R global optimisation package deoptim (see Fig S2).

The parameters related to cell differentiation (ϵ^{QQ}, ϵ^{NQ}, ϵ^{Q}) were chosen to match the starvation dynamics observed in the laboratory: the fact that Q and NQ cells appear within ~4 days after the resource is depleted and that after 4 days of the batch culture Q cells constitute ~ 75% of the population (Fig S1).

We note that the results from the model are robust and altering parameter values only changes the results quantitatively but not qualitatively.

The parameter related to nutrient recycling (r) does influence the system behaviour, and its effect is explored in our simulations as it differs for the H20 (r-0) and YPD (r=0.5) cultures.

**Parameter name**	**Value**	**comments**
V	130 [mmol glucose / g x h]	fitted to data from fresh cell cultures
K	91 [mmol glucose]	fitted to data from fresh cell cultures
n_e^G	0.007 [g. /mmol glucose]	fitted data from fresh cell cultures
t_{lag}	see Supplementary Table 1 and Fig. 5	Found experimentally: different for each experimental setup
d_N	0.005 [1/h]	fitted to represent the starvation dynamics, based on Allen et al. 2006
d_Q	0.002 [1/h]	fitted to represent the starvation dynamics, based on Allen et al. 2006
ϵ^{QQ}	0.25 [1/h]	Chosen to represent the dynamics observed in the laboratory and represented in Fig S1<br><br>Results are robust to variations in this parameter
ϵ^{NQ}	0.2 [1/h]	Chosen to represent the dynamics observed in the laboratory and represented in Fig S1<br><br>Results are robust to variations in this parameter
ϵ^{Q}	0.02 [1/h]	Chosen to represent the dynamics observed in the

		laboratory and represented in Fig S1<br><br>Results are robust to variation in this parameter
ϵ^{AG}	0.0001 [1/h]	Results are robust to variations in this parameter
r	0 for H20, 0.5 for YPD	The dependence of the results on this parameter is explored in this study
η^{Q}	0.9	Results do not change significantly if we assume $\eta^{Q} = 1$.<br><br>Low values of η^{Q} $(\eta^{Q} \sim 0)$ would represent an unrealistic scenario in which the transition into the Q state requires a lot of energy and therefore a significant part of the biomass is lost from the system.

Table S3: Default model parameters

References

1. Allen C et al. 2006 Isolation of quiescent and nonquiescent cells from yeast stationary-phase cultures. J. Cell Biol. 174, 89–100. (doi:10.1083/jcb.200604072)
2. Lee H-Y, Cheng K-Y, Chao J-C, Leu J-Y. 2016 Differentiated cytoplasmic granule formation in quiescent and non-quiescent cells upon chronological aging. Microb. Cell 3, 109–119. (doi:10.15698/mic2016.03.484)
